# Rab11-FIP1 and Rab11-FIP5 Regulate pIgR/pIgA Transcytosis through TRIM21-Mediated Polyubiquitination

**DOI:** 10.3390/ijms221910466

**Published:** 2021-09-28

**Authors:** Xuxu Fan, Dihan Zhou, Bali Zhao, Huijun Sha, Mengxue Li, Xian Li, Jingyi Yang, Huimin Yan

**Affiliations:** 1 Mucosal Immunity Research Group, State Key Laboratory of Virology, Wuhan Institute of Virology, Center for Biosafety Mega-Science, Chinese Academy of Sciences, Wuhan 430071, China; xxfan0803@163.com (X.F.); words1846@hotmail.com (D.Z.); huijunsha2021@163.com (H.S.); lmx22001144@163.com (M.L.); lixian_95724@163.com (X.L.); 2 University of Chinese Academy of Sciences, Beijing 100049, China; 3 Vaccine and Immunology Research Center, Translational Medical Research Institute, Shanghai Public Health Clinical Center, Fudan University, Shanghai 201508, China; zhao.bali@163.com

**Keywords:** pIgR, pIgA, transcytosis, Rab11-FIP1, Rab11-FIP5, TRIM21

## Abstract

Polymeric immunoglobulin receptor (pIgR)-mediated polymeric immunoglobulin A (pIgA) transcytosis across mucosal epithelial cells plays an essential role in mucosal immunity. The general trafficking process has been well investigated, yet the elaborate regulatory mechanisms remain enigmatic. We identified a new pIgR interacting protein, the Rab11 effector Rab11-FIP1. Rab11-FIP1 and Rab11-FIP5 knockdown additively impaired pIgA transcytosis in both polarized and incompletely polarized cells. Moreover, Rab11-FIP1 and Rab11-FIP5 knockdown exhibited more significant inhibitory effects on pIgA transcytosis in incompletely polarized cells than in polarized cells. Interestingly, the trafficking process of pIgA in incompletely polarized cells is distinct from that in polarized cells. In incompletely polarized cells, the endocytic pIgR/pIgA was first transported from the basolateral plasma membrane to the vicinity of the centrosome where Rab11-FIP1 and Rab11-FIP5 bound to it, before the Rab11a-positive endosomes containing pIgR/pIgA, Rab11-FIP1 and Rab11-FIP5 were further transported to the apical plasma membrane via Golgi apparatus. During the trafficking process, TRIM21 mediated the K11-linked polyubiquitination of Rab11-FIP1 and the K6-linked polyubiquitination of Rab11-FIP5 to promote their activation and pIgA transcytosis. This study indicates that polyubiquitinated Rab11-FIP1 and Rab11-FIP5 mediated by TRIM21 cooperatively facilitate pIgA transcytosis and provides new insights into the intracellular trafficking process of pIgA in incompletely polarized cells.

## 1. Introduction

The mucosal immune system is the critical first line of the body to resist invasion of multiple pathogens, such as bacteria, viruses, and parasites [1,2]. Polymeric immunoglobulin A (pIgA), a predominant mucosal antibody, is recognized and bound by the polymeric immunoglobulin receptor (pIgR) [3,4]. The pIgR–pIgA complex is further transported across mucosal epithelial cells and secreted at mucosal surfaces [5]. Secretory IgA (sIgA) plays significant immunoprotective roles at mucosal surfaces to defend against inhaled or ingested pathogens and to confine commensal bacteria to the intestinal lumen [6]. In polarized epithelial cells, pIgR is first delivered to the basolateral plasma membrane of epithelial cells where it binds to pIgA. Subsequently, the pIgR–pIgA complex is endocytosed through clathrin-dependent coat vesicles and traversed sequentially from the basolateral early endosome (BEE) to common recycling endosome (CRE) and further to Rab11a-positive apical recycling endosome (ARE). The endosome is eventually delivered to the apical plasma membrane where pIgR is proteolytically cleaved to yield a secretory component (SC), after which this form of the receptor or sIgA is secreted [7,8,9]. The general trafficking process is well known to us, but the elaborate regulatory mechanism needs to be further explored.

The Rab family of small GTPases and their effectors collaboratively play roles during the different stages of cargo trafficking, such as the endocytosis of vesicles, fusion of vesicles with organelles and exocytosis of vesicles [10,11]. Rab11a, an extensively studied member of the Rab family, is a recycling endosome marker but is also found in the early endosomes and Golgi apparatus [12,13,14]. The location of Rab11a is dynamically changed to regulate multiple cellular processes, such as endocytic recycling, secretion, and cytokinesis [15]. Rab11a serves as an ARE marker, and destruction of the ARE structure impairs the trafficking of pIgA across polarized mucosal epithelial cells [16]. Rab11 family interacting proteins (Rab11-FIPs), Rab11 effectors, are involved in the trafficking of vesicles from the endosomal recycling compartment (ERC) to plasma membrane, receptor-mediated endocytosis, and membrane trafficking of recycling endosomes. Rab11-FIPs are divided into class I and II. Class I Rab11-FIPs are composed of three members: Rab11-FIP1, Rab11FIP2 and Rab11-FIP5 [17,18,19]. Rab11-FIP1 and Rab11-FIP5 bind to Rab11a to facilitate vesicle budding and trafficking. Recently, it has been reported that Rab11-FIP5 plays a crucial role in pIgA transcytosis. During this process, Rab11-FIP5 can be activated by phosphorylation at multiple serine sites [20]. Moreover, Rab11-FIP1 has been shown to regulate the trafficking of multiple cargoes, such as transferrin, integrin, and receptor tyrosine kinase (RTK) in eukaryotic cells [21,22,23]. However, it remains unclear whether Rab11-FIP1 participates in pIgA transcytosis.

Tripartite motif-containing 21 (TRIM21) is an interferon-inducible E3 ligase that plays a crucial role in antiviral innate immunity [24]. TRIM21 promotes K27-linked polyubiquitination of mitochondrial antiviral signaling protein (MAVS) to positively regulate the innate immune response [25]. In addition, TRIM21 serves as a cytosolic Fc receptor that recognizes and binds the three most prevalent circulatory antibodies, namely, IgA, IgM, and IgG [6]. Antibodies attached to pathogens can be carried inside cells during infection. Once inside the cytosol, antibody-bound viruses are rapidly recognized by TRIM21 [26]. TRIM21 catalyzes the formation of K48-linked ubiquitin moieties to degrade incoming viruses via the proteasome and AAA ATPase valosin-containing protein (VCP), simultaneously activating effective intracellular virus neutralization and innate immune signaling to inhibit virus replication [27]. However, the role of TRIM21 in pIgR–pIgA transcytosis needs to be further clarified.

In this study, we found that Rab11-FIP1 and Rab11-FIP5 could interact with pIgR and that knockdown of Rab11-FIP1 and Rab11-FIP5 significantly inhibited pIgA transcytosis. In addition, in incompletely polarized cells, the endocytic pIgR–pIgA complex was first transported from the basolateral plasma membrane to the vicinity of centrosome where Rab11-FIP1 and Rab11-FIP5 formed a complex. During the trafficking process, TRIM21 mediated the K11-linked polyubiquitination of Rab11-FIP1 and the K6-linked polyubiquitination of Rab11-FIP5 to promote their activation. With the assistance of Rab11-FIP1 and Rab11-FIP5, the pIgR–pIgA complex was further transferred to the apical plasma membrane via Golgi apparatus and then secreted. Our results provide new insights into the intracellular trafficking mechanisms of pIgA.

## 2. Results

### 2.1. Rab11-FIP1 Interacts with pIgR

**Figure 1:**
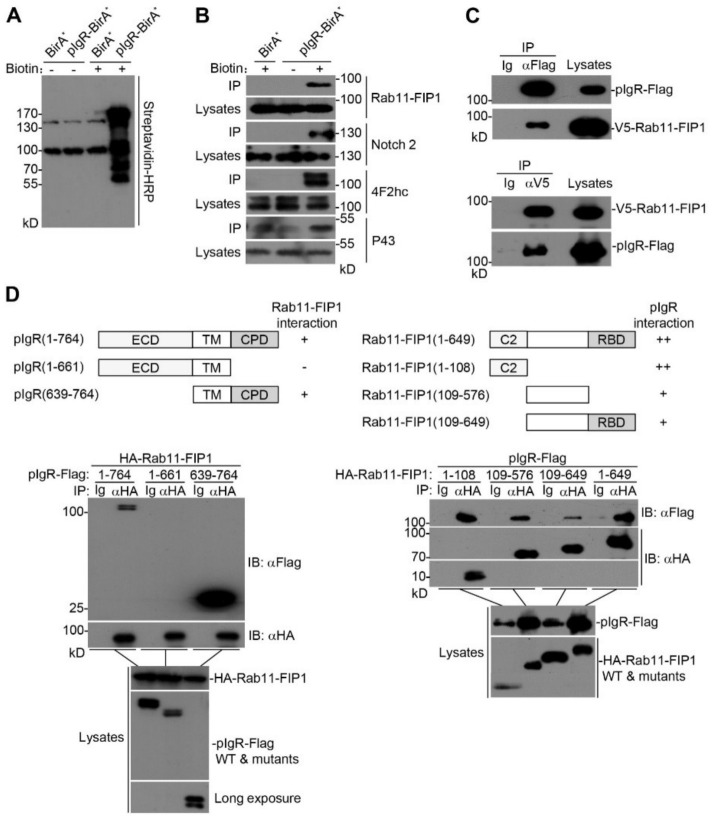
Rab11−FIP1 interacts with pIgR. (**A**) HEK293T cells (2 × 10^6^) were transfected with pIgR−BirA* or BirA* plasmids for 24 h. Cells were untreated or treated with 50 µM biotin for 12 h before lysis in the indicated buffer. Biotin−labelled proteins were immunoprecipitated from lysates using streptavidin conjugated agarose beads. Immunoprecipitated proteins were detected by immunoblotting analysis and identified by mass spectrometry. (**B**) The samples of (**A**) were respectively detected by immunoblotting analysis with the indicated antibodies. (**C**) Detection of the interaction between Rab11−FIP1 and pIgR. HEK293T cells (2 × 10^6^) were co−transfected with the indicated plasmids for 24 h. Coimmunoprecipitation and immunoblotting analyses were performed with the indicated antibodies. (**D**) Domain mapping of the interaction between Rab11−FIP1 and pIgR. HEK293T cells (2 × 10^6^) were co−transfected with the indicated plasmids for 24 h. Coimmunoprecipitation and immunoblotting analyses were performed with the indicated antibodies. ECD: ectodomain; TM: transmembrane domain; CPD: cytoplasmic domain. ++: stronger interaction, +: interaction, −: no interaction. Data of (**A**–**D**) are representative of three independent experiments.

To further unveil the process of pIgR–pIgA transcytosis and the underlying mechanisms, proximity-dependent biotinylation (BioID) and mass spectrometry methods were performed to identify potential molecules participating in pIgR–pIgA transcytosis [28]. To this end, we first inserted pIgR cDNA into a vector containing the BirA R118G gene (BirA*), which expresses a DNA-binding biotin protein ligase. Subsequently, HEK293T cells expressing pIgR-BirA* were incubated with or without biotin, and then streptavidin agarose beads were used to enrich the biotin-labeled proteins. Immunoblotting analysis showed that multiple enriched proteins might be able to interact with pIgR, which were further identified by mass spectrometry (Figure 1A and Supplementary Table S1). Some of the identified proteins were verified by immunoblotting analysis with the corresponding antibodies (Figure 1B). Rab11-FIP1, an effector protein of Rab11 that regulates the recycling of integrin and other receptors, was one of the identified candidate proteins interacting with pIgR. Therefore, we focused on the relationship between Rab11-FIP1 and pIgR. Coimmunoprecipitation experiments confirmed that Rab11-FIP1 interacted with pIgR (Figure 1C). Domain mapping experiments indicated that the cytoplasmic domain (CPD) of pIgR was essential for its interaction with Rab11-FIP1. In addition, the C2 domain of Rab11-FIP1 played a predominant role in binding with pIgR (Figure 1D). These results suggest that Rab11-FIP1 could interact with pIgR.

### 2.2. Rab11-FIP1 Colocalizes with pIgA during pIgA Transcytosis

**Figure 2:**
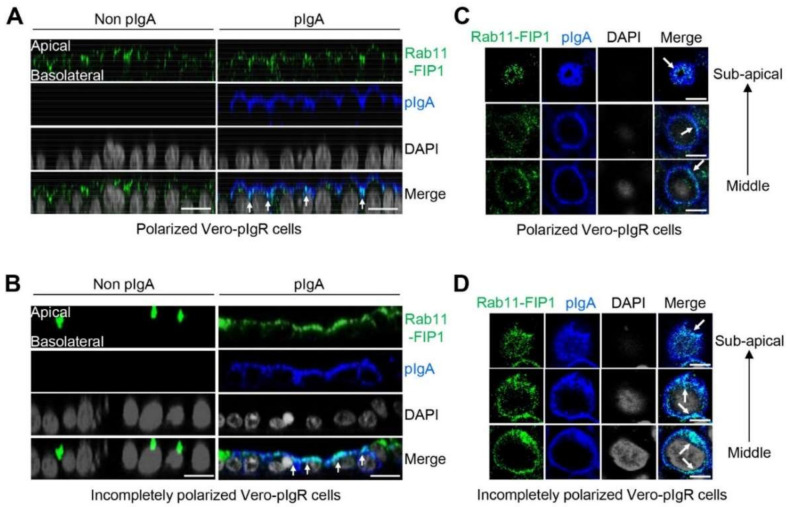
Rab11−FIP1 colocalizes with pIgA during pIgA transcytosis. (**A**,**B**) Analysis of Rab11−FIP1 distribution in polarized and incompletely polarized cells. Vero−pIgR cells (1 × 10^5^) were grown on 12 mm diameter Transwell (0.4 µm pore) for 6 days (**A**). Vero−pIgR cells (1 × 10^5^) were grown on 12 mm diameter Transwell (0.4 µm pore) for 3 days (**B**). An amount of 20 µg pIgA was added or not added to the basal chamber for 1 h. The cells were fixed with 4% paraformaldehyde and stained with the indicated antibodies before observation by confocal microscopy. Scale bar: 20 µm. (**C**,**D**) Analysis of Rab11−FIP1 and pIgA distribution at different regions of Vero−pIgR cells. Vero−pIgR cells (1 × 10^5^) were grown on Transwell for 6 days (**C**). Vero−pIgR cells (1 × 10^5^) were grown on 12 mm diameter Transwell (0.4 µm pore) for 3 days (**D**). An amount of 20 µg pIgA was added to the basal chamber for 1 h. Subsequently, cells were fixed with 4% paraformaldehyde and stained with the indicated antibodies before observation by confocal microscopy. Cells in the same field of vision were successively observed from the middle region to subapical region. Scale bar: 10 µm. Data of (**A**–**D**) are representative of three independent experiments.

Previous studies have shown that pIgR binds pIgA at the basolateral surface of mucosal epithelial cells and mediates pIgA transcytosis [8]. The endocytic pIgR–pIgA complex is then transported across the cells for secretion at the apical surface [29]. In line with the previous report, confocal microscopy showed that pIgA was strictly colocalized with pIgR (Supplementary Figure S1A). As we found Rab11-FIP1 interacted with pIgR, we next investigated whether Rab11-FIP1 was involved in the transcytosis of pIgA. Transwell system is a classic model to study pIgR-mediated pIgA transcytosis in polarized epithelial cells [30]. In this study, Transwell was also used to culture incompletely polarized cells with monolayer integrity, but without macromolecular permeability. The transepithelial resistance (TER) in Transwell system was measured every day to indicate monolayer polarization in Vero cells stably expressing pIgR (Vero-pIgR) or Caco-2 cells (Supplementary Figure S2A,B). As shown in Figure 2A and Figure S1B, the vertical cross-sectional (XZ-plane) images revealed that Rab11-FIP1 was mainly distributed in the apical domain of polarized Vero-pIgR or Caco-2 cells, regardless of whether pIgA was transported. Additionally, Rab11-FIP1 partially colocalized with pIgA during its intracellular transcytosis across these polarized cell types. Interestingly, we found that the distribution of Rab11-FIP1 in incompletely polarized Vero-pIgR cells was markedly different from that in polarized Vero-pIgR cells in the absence of pIgA. As depicted in Figure 2B, Rab11-FIP1 mainly aggregated in the perinuclear compartment of incompletely polarized Vero-pIgR cells without pIgA transcytosis, whereas it was dispersed and partially colocalized with pIgA during pIgA transcytosis. The distinct distribution of Rab11-FIP1 in incompletely polarized Vero-pIgR cells drove us to further explore its dynamic interaction with pIgR–pIgA complex in detail. In addition, because the low expression of pIgR results in limited transport efficiency of pIgA in Caco-2 cells, we further explored the above phenomenon in Vero-pIgR cells [7].

To carefully observe the colocalization of Rab11-FIP1 and pIgA during pIgA transcytosis, the cells in the same field of vision were successively observed from the middle region to the subapical region, and we found that Rab11-FIP1 always colocalized with pIgA during pIgA trafficking through these sites (Figure 2C,D). These results suggest that Rab11-FIP1 colocalizes with pIgA during pIgA trafficking in both incompletely polarized and polarized Vero-pIgR cells.

### 2.3. Rab11-FIP1 and Rab11-FIP5 Additively Promote Extracellular Secretion of pIgA

**Figure 3:**
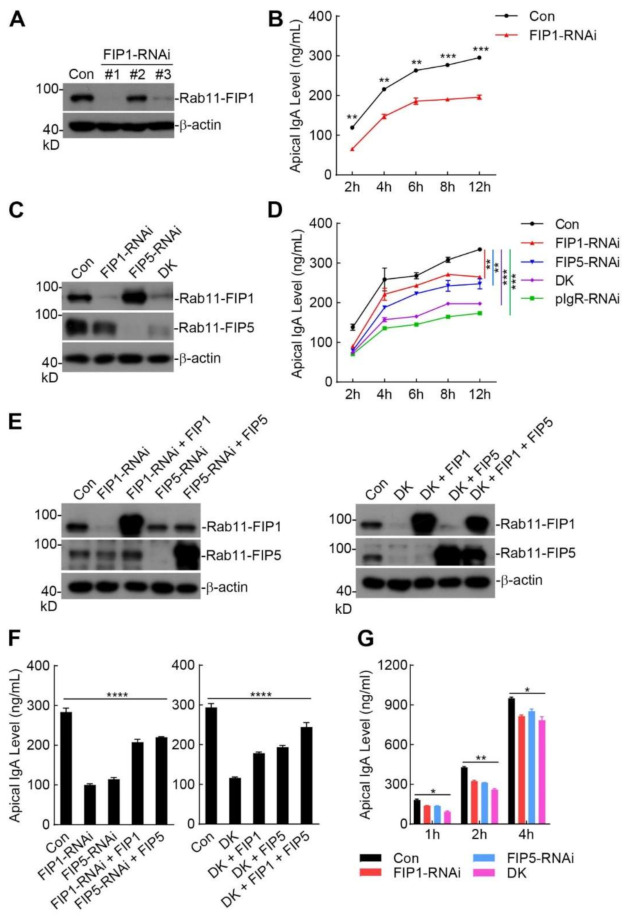
Knockdown of Rab11−FIP1 or Rab11−FIP5 impairs extracellular secretion of pIgA. (**A**,**B**) Effects of Rab11−FIP1 knockdown on extracellular secretion of pIgA. (**A**) Vero−pIgR cells were transduced with control (Con) or the indicated Rab11−FIP1−RNAi plasmids. The knockdown efficiencies were detected by immunoblotting analysis. (**B**) The control and Rab11−FIP1−RNAi Vero−pIgR cells (1 × 10^5^) (Rab11−FIP1−RNAi #1 plasmid was used) were grown on Transwell for 3 days. An amount of 20 µg pIgA was added to the basal chamber for the indicated time points. The supernatant was collected for IgA analysis by ELISA. (**C**,**D**) Effects of double knockdown of Rab11−FIP1 and Rab11−FIP5 on extracellular secretion of pIgA. (**C**) Vero−pIgR cells were transduced with the control or indicated RNAi plasmids by retroviral−mediated gene transfer to establish stable cell lines. The knockdown efficiencies of these genes in their cell lines were detected by immunoblotting analysis. (**D**) The indicated Vero−pIgR cells (1 × 10^5^) were grown on Transwell for 3 days. An amount of 20 µg pIgA was added to the basal chamber for the indicated time points. The supernatant was collected for IgA analysis by ELISA. DK: double knockdown. (**E**,**F**) Effects of reconstitution of Rab11−FIP1 knockdown, Rab11−FIP5 knockdown, Rab11−FIP1 and Rab11−FIP5 double-knockdown cells with Rab11−FIP1 or Rab11−FIP5 plasmids on extracellular secretion of pIgA. (**E**) The indicated knockdown Vero−pIgR cell lines were transduced with the indicated plasmids. The knockdown and reconstitution efficiencies of these proteins were detected by immunoblotting analysis. (**F**) The indicated Vero−pIgR cell lines (1 × 10^5^) in (**E**) were grown on Transwell for 3 days. An amount of 20 µg pIgA was added to the basal chamber for 4 h. The supernatant was collected for IgA analysis by ELISA. DK: double knockdown. (**G**) The Vero−pIgR cells (1 × 10^5^) were grown on Transwell for 6 days. An amount of 20 µg pIgA was added to the basal chamber for the indicated time points. The supernatant was collected for IgA analysis by ELISA. Data of (**B**,**D**) were analyzed by unpaired, two−tailed Student’s *t*−test. Data of (**F**,**G**) were analyzed by Kruskal−Wallis one−way ANOVA. Graphs show mean ± SD; *n* = 3. * *p* < 0.05, ** *p* < 0.01, *** *p* < 0.001, **** *p* < 0.0001. Data of (**A**–**G**) are representative of three independent experiments. FIP1 was abbreviated form of Rab11−FIP1, FIP5 was abbreviated form of Rab11−FIP5.

Next, we investigated the effects of Rab11-FIP1 on the extracellular secretion of pIgA. As depicted in Figure 3A, we constructed three RNAi plasmids that specifically targeted the coding sequence of the Rab11-FIP1 gene and dramatically downregulated the expression of Rab11-FIP1 except Rab11-FIP1-RNAi #2. Enzyme-linked immunosorbent assay (ELISA) experiments suggested markedly decreased levels of sIgA in the apical supernatant when Rab11-FIP1 was downregulated (Figure 3B). Rab11-FIP5, another member of class I Rab11-FIPs, has been reported to participate in pIgR–pIgA transcytosis in MDCK cells [20]. Hence, we further investigated the roles of Rab11-FIP1 and Rab11-FIP5 in the transcytosis and extracellular secretion of pIgA. Stable cell lines with individual or double knockdown of Rab11-FIP1 and Rab11-FIP5 were constructed, with a pIgR-downregulated cell line serving as a positive control (Figure 3C and Figure S3A). As indicated in Figure 3D, double knockdown of Rab11-FIP1 and Rab11-FIP5 inhibited extracellular secretion of pIgA more dramatically than did the individual knockdown of Rab11-FIP1 or Rab11-FIP5. Most importantly, the effects of double knockdown of Rab11-FIP1 and Rab11-FIP5 on extracellular secretion of pIgA were similar to those of pIgR knockdown. Moreover, immunoblotting analysis demonstrated that pIgR knockdown attenuated the intracellular levels of pIgA but that double knockdown of Rab11-FIP1 and Rab11-FIP5 resulted in intracellular accumulation of pIgA (Supplementary Figure S3B,C). These results suggest that Rab11-FIP1 and Rab11-FIP5 collaboratively promote extracellular secretion of pIgA.

To further verify the positive effects of Rab11-FIP1 and Rab11-FIP5 on pIgA transcytosis, Rab11-FIP1 and Rab11-FIP5 individual and double knockdown cells were transduced with the control or corresponding plasmids for reconstitution experiments. As shown in Figure 3E,F, knockdown of Rab11-FIP1 or Rab11-FIP5 attenuated extracellular secretion of pIgA, and reconstitution of the knockdown cells with Rab11-FIP1 or Rab11-FIP5 restored extracellular secretion of pIgA. Moreover, double knockdown of Rab11-FIP1 and Rab11-FIP5 markedly inhibited extracellular secretion of pIgA, and reconstitution of the double knockdown cells with either Rab11-FIP1 or Rab11-FIP5 restored extracellular secretion of pIgA, which was further enhanced by their coreconstitution. As depicted in Figure 3G, Rab11-FIP1 and Rab11-FIP5 knockdown also inhibited the extracellular secretion of pIgA in polarized Vero-pIgR cells. These results suggest that Rab11-FIP1 and Rab11-FIP5 additively facilitate pIgA extracellular secretion. Because Rab11-FIP1 and Rab11-FIP5 knockdown showed a more significant inhibitory effect on pIgA transcytosis in incompletely polarized cells than in polarized cells, we further investigated the regulatory mechanism of pIgA transcytosis in incompletely polarized cells.

### 2.4. Rab11-FIP1, Rab11-FIP5 and pIgR Form a Complex during pIgA Transcytosis

**Figure 4:**
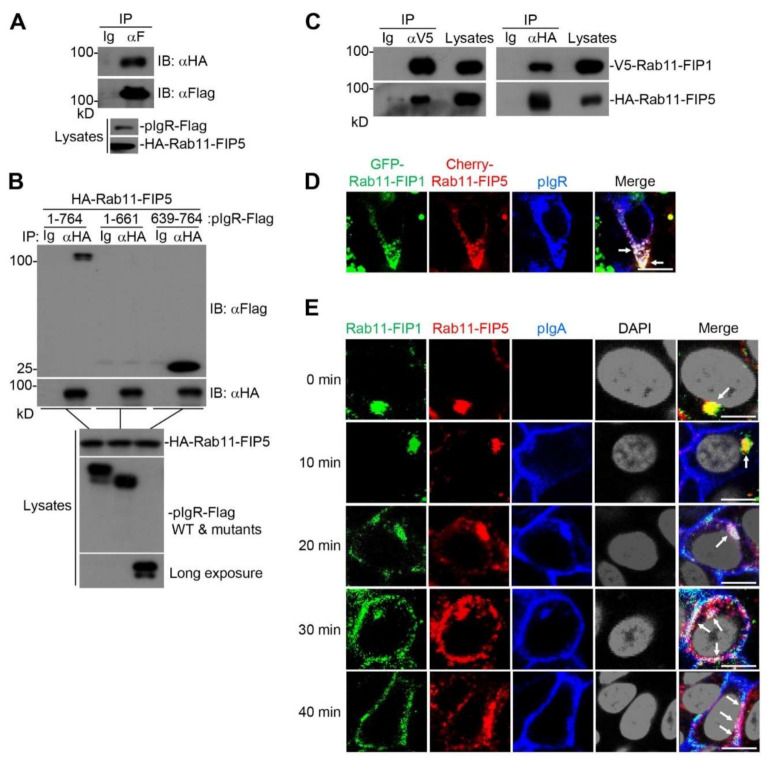
The Rab11−FIP1, Rab11−FIP5 and pIgR complex facilitates pIgA transcytosis. (**A**) The interaction between Rab11−FIP5 and pIgR was detected. HEK293T cells (2 × 10^6^) were co−transfected with the indicated plasmids for 24 h. Coimmunoprecipitation and immunoblot analyses were performed with the indicated antibodies. (**B**) Domain mapping of the interaction between Rab11−FIP5 and pIgR. HEK293T cells (2 × 10^6^) were co−transfected with the indicated plasmids for 24 h. Coimmunoprecipitation and immunoblot analysis were performed with the indicated antibodies. (**C**) The interaction between Rab11−FIP1 and Rab11−FIP5 was detected. HEK293T cells (2 × 10^6^) were co−transfected with the indicated plasmids for 24 h. Coimmunoprecipitation and immunoblotting analyses were performed with the indicated antibodies. (**D**) Colocalization of Rab11−FIP1, Rab11−FIP5 and pIgR was detected. Vero−pIgR cells (1 × 10^5^) were co−transfected with GFP−Rab11−FIP1 (0.2 µg) and Cherry−Rab11−FIP5 (0.2 µg) for 24 h. The transfected cells were fixed with 4% paraformaldehyde and stained with the indicated antibodies before observation by confocal microscopy. Scale bar: 10 µm. (**E**) Colocalization of Rab11−FIP1, Rab11−FIP5 and pIgA was detected during pIgA transcytosis. Vero−pIgR cells (1 × 10^5^) were grown on Transwell for 3 days. An amount of 20 µg pIgA was added or not added to the basal chamber for 10 min at 37 °C and cells were then washed for three times. Subsequently, cells were cultivated at 37 °C and harvested at the indicated time points. Finally, the cells were fixed with 4% paraformaldehyde and stained with the indicated antibodies before observation by confocal microscopy. Scale bar: 10 µm. Data of (**A**–**E**) are representative of three independent experiments.

We next investigated the potential mechanism underlying how Rab11-FIP1 and Rab11-FIP5 additively promote extracellular secretion of pIgA. Coimmunoprecipitation experiments revealed that Rab11-FIP5 was also associated with the full-length pIgR (Figure 4A). Domain mapping experiments indicated that the cytoplasmic domain (CPD) of pIgR was also essential for its interaction with Rab11-FIP5 (Figure 4B). In addition, Rab11-FIP1 was able to interact with Rab11-FIP5 (Figure 4C). As shown in Supplementary Figure S4A, co-transfection of HA-Rab11-FIP5 with V5-Rab11-FIP1 and pIgR-Flag had no effect on the quality of V5-Rab11-FIP1 binding to pIgR-Flag. This result indicates that Rab11-FIP1 and Rab11-FIP5 might not compete to bind pIgR. Confocal microscopy experiments further indicated that Rab11-FIP1, Rab11-FIP5 and pIgR colocalized with each other in the mammalian overexpression system (Figure 4D). Reconstitution of truncation HA-Rab11-FIP1-C2 could not restore extracellular secretion of pIgA and reconstitution of truncation HA-Rab11-FIP5-C2 could partially restore this process (Supplementary Figure S3D,E). Therefore, the full lengths of Rab11-FIP1 and Rab11-FIP5 were important for pIgA transcytosis. As illustrated in Figure 4E, during pIgA transcytosis, Rab11-FIP1 and Rab11-FIP5 aggregated in the perinuclear compartment of incompletely polarized Vero-pIgR cells within the first ~10 min, and were gradually translocated to the cytoplasm after ~20 min. Interestingly, pIgA was mainly located at the basolateral plasma membrane at ~10 min, and a portion of it was translocated and aggregated in the perinuclear compartment at ~20 min. Subsequently, pIgA aggregated in the perinuclear compartment was gradually transferred to the apical plasma membrane after ~20 min. During pIgA transcytosis, pIgA did not colocalize with Rab11-FIP1 and Rab11-FIP5 within the first ~10 min, and a fraction of pIgA colocalized with them at ~20 min. Moreover, colocalization of pIgA with the two proteins was more significant after ~20 min (Supplementary Figure S4B). Consistently, endogenous Rab11-FIP1 and Rab11-FIP5 also colocalized with each other in the perinuclear compartment of incompletely polarized Caco-2 cells without pIgA transcytosis (Supplementary Figure S4C). These results indicate that Rab11-FIP1, Rab11-FIP5 and pIgR serve as a complex to additively promote pIgA transcytosis.

### 2.5. Rab11-FIP1 Colocalizes with the Transcytosed pIgA from the Vicinity of Centrosome via Golgi Apparatus to Apical Plasma Membrane in Incompletely Polarized Vero-pIgR Cells

**Figure 5:**
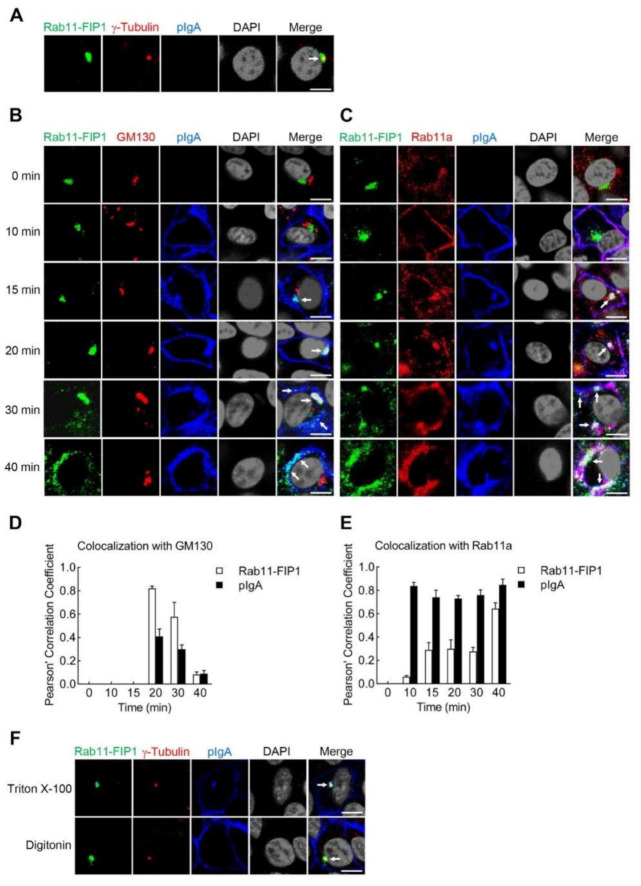
Rab11−FIP1 colocalizes with pIgA during pIgA transcytosis from the vicinity of centrosome to apical plasma membrane. (**A**) Colocalization of Rab11−FIP1 with the centrosome was detected. Vero−pIgR cells (1 × 10^5^) were grown on Transwell for 3 days. Cells were fixed with 4% paraformaldehyde and stained with the indicated antibodies before observation by confocal microscopy. Scale bar: 10 µm. (**B**,**C**) Colocalization of Rab11−FIP1, pIgA with the Golgi apparatus (GM130) or recycling endosomes (Rab11a) was detected. Vero−pIgR cells (1 × 10^5^) were grown on Transwell for 3 days. An amount of 20 µg pIgA was added or not added to the basal chamber for 10 min at 37 °C and cells were then washed three times. Subsequently, cells were cultivated at 37 °C and harvested at the indicated time points. Finally, cells were fixed with 4% paraformaldehyde and stained with the indicated antibodies before observation by confocal microscopy. Scale bar: 10 µm. (**D**,**E**) Quantitative analysis of colocalization of Rab11−FIP1, pIgA with GM130 or Rab11a. Statistical analysis was based on colocalization images (covering dozens of cells) using the ImageJ software. (**F**) Analysis of localization of Rab11−FIP1 on the endosomes containing pIgA. Vero−pIgR cells (1 × 10^5^) were grown on Transwell for 3 days. An amount of 20 µg pIgA was added to the basal chamber for 15 min at 37 °C and cells were then washed three times. Cells were fixed with 4% paraformaldehyde and then were permeabilized by 0.1% Triton X−100 or 20 µg/mL digitonin for 10 min at 4 °C. Finally, cells were stained with the indicated antibodies before observation by confocal microscopy. Scale bar: 10 µm. Data of (**A**–**F**) are representative of three independent experiments.

The trafficking process of pIgA in polarized mucosal epithelial cells has been well investigated [7,8,9]. As depicted in Figure 2A,B, the distribution of Rab11-FIP1 in incompletely polarized Vero-pIgR cells was markedly different from that in polarized Vero-pIgR cells in the absence of pIgA. In addition, as shown in Figure 3D–G, Rab11-FIP1 and Rab11-FIP5 knockdown showed a more significant inhibitory effect on pIgA transcytosis in incompletely polarized cells than in polarized cells. These results drove us to further explore the trafficking process of pIgA in incompletely polarized cells, and the underlying regulatory mechanisms in the participation of Rab11-FIP1 and Rab11-FIP5 in pIgA transcytosis. Hence, confocal microscopy experiments were performed to investigate the detailed trafficking process of pIgA and Rab11-FIP1 in incompletely polarized Vero-pIgR cells. Endogenous markers for the centrosome (γ-Tubulin), Golgi apparatus (GM130), recycling endosomes (Rab11a), and endoplasmic reticulum (ER) (KDEL) were stained. As shown in Figure 5A, Rab11-FIP1 colocalized with the centrosome in incompletely polarized Vero-pIgR cells without pIgA treatment, and it also aggregated in the centrosome of incompletely polarized Caco-2 cells (Supplementary Figure S4D). As shown in Figure 5B, neither Rab11-FIP1 nor pIgA colocalized with the Golgi apparatus within the first ~15 min following pIgA treatment. Rab11-FIP1 and pIgA colocalized in the vicinity of centrosome at ~15 min. Subsequently, the recycling endosomes containing Rab11-FIP1 and pIgA were translocated to the Golgi apparatus together at ~20 min, and migrated away from the Golgi apparatus and further transferred to the apical plasma membrane after ~20 min. These results suggest that the recycling endosomes containing Rab11-FIP1 and pIgA were sequentially translocated from the vicinity of centrosome to Golgi apparatus and further to apical plasma membrane during pIgA transcytosis. As illustrated in Figure 5C, Rab11a was diffusely distributed in the cytoplasm without pIgA treatment. Following pIgA treatment, Rab11a rapidly moved towards the basolateral plasma membrane and colocalized with pIgA within ~10 min. Subsequently, Rab11a-positive endosomes always colocalized with pIgA when it traversed from the basolateral plasma membrane to apical plasma membrane. These results indicate that Rab11a-positive endosomes participated in the whole process of pIgA trafficking. As shown in Supplementary Figure S5, neither Rab11-FIP1 nor pIgA colocalized with the ER during pIgA transcytosis. In addition, quantitative analysis of colocalization of Rab11-FIP1 and pIgA with GM130 or Rab11a confirmed the above results (Figure 5D,E).

To further investigate whether the interaction between Rab11-FIP1 and pIgR occurred in the cytosolic or luminal side of Rab11a-positive endosomes, we performed the following experiments. Triton X-100 permeabilizes the membrane including plasma membrane and organelles membrane. Digitonin has the ability to permeabilize the plasma membrane, while leaving intracellular organelles intact [31,32]. Therefore, as a ligand for pIgR, pIgA would reside on the luminal side of recycling endosomes and thus could only be stained after permeabilization with Triton X-100 but not digitonin. As shown in Figure 5F, Rab11-FIP1 and pIgA were stained and colocalized in the vicinity of centrosome at ~15 min following pIgA transcytosis in incompletely polarized Vero-pIgR cells when treated with Triton X-100. Interestingly, upon digitonin treatment, only Rab11-FIP1 could be stained and localized in the centrosome, which indicates that Rab11-FIP1 and pIgR colocalized in the cytosolic side of recycling endosomes. Taken together, these results suggest that Rab11a-positive endosomes containing Rab11-FIP1-pIgA traverse sequentially from the vicinity of centrosome via Golgi apparatus to apical plasma membrane during pIgA transcytosis in incompletely polarized Vero-pIgR cells.

### 2.6. Polyubiquitinated Rab11-FIP1 and Rab11-FIP5 Mediated by TRIM21 Regulate pIgA Transcytosis

**Figure 6:**
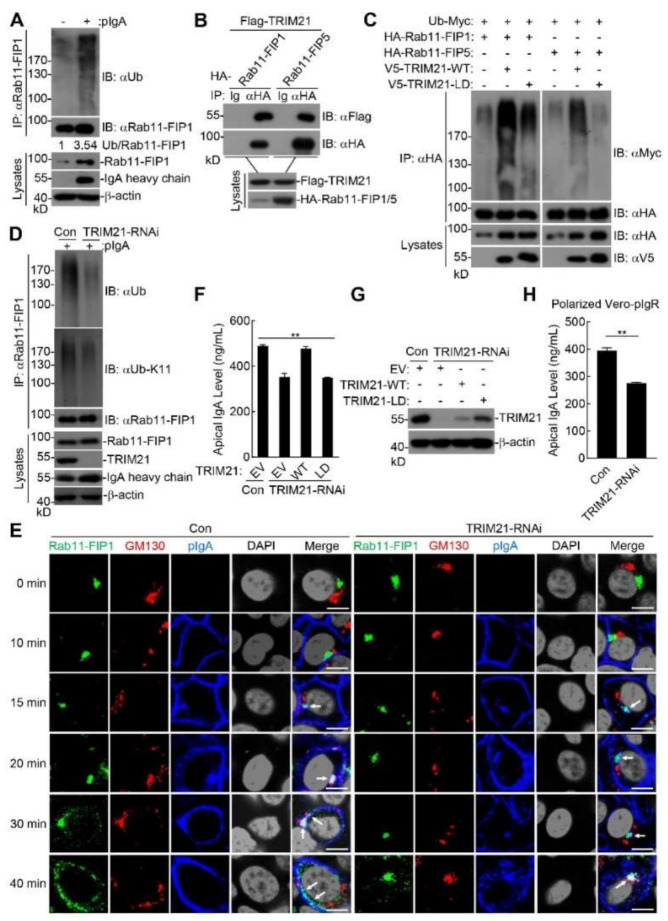
TRIM21−mediated polyubiquitination of Rab11−FIP1 and Rab11−FIP5 is crucial for their ability to facilitate pIgA transcytosis. (**A**) Effects of pIgA transcytosis on the ubiquitination of Rab11−FIP1. Vero−pIgR cells (4 × 10^5^) were grown on 24 mm diameter Transwell (0.4 µm pore) for 3 days. An amount of 80 µg pIgA was added or not added to the basal chamber for 1 h. The cells (2 × 10^7^) were harvested for ubiquitination assays with the indicated antibodies. (**B**) The interaction between Rab11−FIP1, Rab11−FIP5 and TRIM21 was detected. HEK293T cells (2 × 10^6^) were co−transfected with the indicated plasmids for 24 h. Coimmunoprecipitation and immunoblotting analyses were performed with the indicated antibodies. (**C**) TRIM21 mediated polyubiquitination of Rab11−FIP1 and Rab11−FIP5 was detected. HEK293T cells (2 × 10^6^) were co−transfected with the indicated plasmids for 24 h. Ubiquitination assays were performed with the indicated antibodies. (**D**) Effects of TRIM21 knockdown on the K11−linked ubiquitination of Rab11−FIP1 during pIgA transcytosis. Vero−pIgR cells were transduced with control or the indicated TRIM21−RNAi plasmids by retroviral−mediated gene transfer to establish stable cell lines. The indicated Vero−pIgR cells (4 × 10^5^) were grown on 24 mm diameter Transwell for 3 days. An amount of 80 µg pIgA was added or not added to the basal chamber for 1 h. The cells (2 × 10^7^) were harvested for ubiquitination assays with the indicated antibodies. (**E**) Effects of TRIM21 knockdown on pIgA transcytosis. The control and TRIM21−RNAi Vero−pIgR cells (1 × 10^5^) were grown on Transwell for 3 days. An amount of 20 µg pIgA was added or not added to the basal chamber for 10 min at 37 °C and cells were then washed three times. Subsequently, cells were cultivated at 37 °C and harvested at the indicated time points. Finally, cells were fixed with 4% paraformaldehyde and stained with the indicated antibodies before observation by confocal microscopy. Scale bar: 10 µm. (**F**) Effects of reconstitution of TRIM21−knockdown cells with TRIM21 but not its LD mutant on extracellular secretion of pIgA. The control and TRIM21−RNAi Vero−pIgR cells transduced with the indicated plasmids were grown on Transwell for 3 days. An amount of 20 µg pIgA was added to the basal chamber for 4 h. The supernatant was collected for IgA analysis by ELISA. (**G**) The knockdown and reconstitution efficiencies of TRIM21 in the cells were detected by immunoblotting analysis. (**H**) Effects of TRIM21 knockdown on extracellular secretion of pIgA in polarized Vero−pIgR cells. The indicated Vero−pIgR cells (1 × 10^5^) were grown on Transwell for 6 days. An amount of 20 µg pIgA was added to the basal chamber for 2 h. The supernatant was collected for IgA analysis by ELISA. Data of (**F**) were analyzed by Kruskal−Wallis one−way ANOVA. Data of (**H**) were analyzed by unpaired, two−tailed Student’s *t*−test. Graphs show mean ± SD; *n* = 3. ** *p* < 0.01. Data of (**A**–**H**) are representative of three independent experiments. EV: empty vector. WT: wild type plasmid. LD: ligase−dead (LD) mutant plasmid.

Since pIgA transcytosis is a tightly regulated process, we next investigated the mechanisms underlying how Rab11-FIP1 and Rab11-FIP5 participated in pIgA transcytosis. We found that the mRNA and protein levels of Rab11-FIP1 and Rab11-FIP5 increased during pIgA transcytosis (Supplementary Figure S6A,B). It has been shown that ubiquitination regulates the entry of cargo proteins into vesicles at different stages of the endocytosis pathway to promote their activation or degradation [33,34,35]. As shown in Figure 6A, during pIgA transcytosis, Rab11-FIP1 underwent polyubiquitination. Coimmunoprecipitation experiments shown in Figure 6B demonstrated that Rab11-FIP1 and Rab11-FIP5 interacted with TRIM21. As TRIM21 is an E3 ubiquitin ligase, we next explored whether TRIM21 ubiquitinates Rab11-FIP1 and Rab11-FIP5. As illustrated in Figure 6C, TRIM21 but not its ligase-dead (LD) mutant (C16A, C31A, and H33W) markedly enhanced polyubiquitination of Rab11-FIP1 and Rab11-FIP5 in the mammalian overexpression system [36,37]. In vitro ubiquitination assays indicated that TRIM21 catalyzed polyubiquitination of Rab11-FIP1 and Rab11-FIP5 with UBCH5b or UBCH5c as an E2 and that TRIM21-LD mutant had little activity in these assays (Supplementary Figure S7A–D). Overexpression of UBCH5c enhanced TRIM21-mediated polyubiquitination of Rab11-FIP1 and Rab11-FIP5 (Supplementary Figure S7E,F). These results suggest that the E3 ligase activity of TRIM21 was required for polyubiquitination of Rab11-FIP1 and Rab11-FIP5. Further experiments with linkage-specific ubiquitin indicated that TRIM21 mediated the K11-linked polyubiquitination of Rab11-FIP1 and the K6-linked polyubiquitination of Rab11-FIP5 (Supplementary Figures S8A,B and S9A,B). Endogenous ubiquitination assays indicated that K11-linked polyubiquitination of Rab11-FIP1 was partially impaired in TRIM21-knockdown cells during pIgA transcytosis (Figure 6D). These results suggest that TRIM21 mediates the K11-linked polyubiquitination of Rab11-FIP1 and the K6-linked polyubiquitination of Rab11-FIP5 during pIgA transcytosis.

As shown in Figure 6E, compared with wild-type cells, pIgA was still transferred from the basolateral plasma membrane to the vicinity of centrosome and colocalized with Rab11-FIP1 at ~15 min in TRIM21-knockdown cells. Nevertheless, Rab11-FIP1-pIgA invisibly colocalized with the Golgi apparatus at ~20 min and translocated to the Golgi apparatus up to ~40 min, indicating that TRIM21 knockdown did not affect pIgA trafficking from the basolateral plasma membrane to the vicinity of centrosome, but did impair trafficking of Rab11-FIP1-pIgA from the vicinity of centrosome to Golgi apparatus and delay pIgA transcytosis. Quantitative analysis of colocalization of Rab11-FIP1 and pIgA with GM130 in wild-type and TRIM21-knockdown cells confirmed the above results (Supplementary Figure S10). In addition, knockdown of TRIM21 attenuated extracellular secretion of pIgA while reconstitution of the knockdown cells with TRIM21 but not its ligase-dead (LD) mutant restored extracellular secretion of pIgA (Figure 6F,G). Furthermore, in polarized Vero-pIgR cells, knockdown of TRIM21 could also attenuate extracellular secretion of pIgA (Figure 6H). These results indicate that TRIM21-mediated polyubiquitination of Rab11-FIP1 and Rab11-FIP5 is crucial for their ability to promote the trafficking of pIgA from the vicinity of centrosome via Golgi apparatus to apical plasma membrane, thereby facilitating extracellular secretion of pIgA.

## 3. Discussion

PIgA is a kind of prevalent antibody type that is continuously transported through mucosal epithelial cells, and its important immunoprotective role has become increasingly clear [38,39]. In polarized mucosal epithelial cells of the respiratory or digestive tracts, the pIgR–pIgA complex is transported from the basolateral plasma membrane to apical plasma membrane via the BEE-CE-ARE pathway [40]. In recent years, some proteins have been reported to regulate pIgA transcytosis in polarized mucosal epithelial cells, such as the retromer complex, tyrosine-protein kinase Yes, Rab11a, Rab11-FIP2 and Rab11-FIP5 [41,42,43,44,45,46]. It has been reported that Rab11a and Rab11-FIP5 are enriched in the ARE and involved in pIgA transcytosis in polarized cells [16,20]. In this study, we identified Rab11-FIP1 as a new pIgR interacting protein. We found Rab11-FIP1 and Rab11-FIP5 could form a complex with pIgR and that knockdown of them markedly inhibited pIgA transcytosis in both polarized and incompletely polarized cells. These results indicate that Rab11-FIP1 and Rab11-FIP5 collaboratively promote pIgA transcytosis. More obvious inhibitory effects and the intriguing trafficking process of Rab11-FIP1-pIgA in incompletely polarized cells prompted us to further explore the regulatory mechanism. We found that endocytic pIgA was first translocated from the basolateral plasma membrane to the vicinity of the centrosome to colocalize with Rab11-FIP1 and Rab11-FIP5, and then was further transported to the apical plasma membrane via Golgi apparatus. We hypothesized that the transport complex or the involved motor proteins might need to interact with certain molecular chaperones or effector proteins, such as Rab11-FIP1 and Rab11-FIP5, or undergo unknown modifications in the Golgi apparatus, such as full glycosylation and phosphorylation, thereby facilitating the efficient trafficking and secretion of pIgA. In previous studies, pIgA was found in the Golgi apparatus, which might support our findings [47,48,49]. The recycling endosomes containing pIgR–pIgA complex were associated with Rab11-FIP1 and Rab11-FIP5 in the vicinity of the centrosome through the cytoplasmic domain of pIgR. Rab11-FIP1, Rab11-FIP5 and pIgR formed a complex that collaboratively promoted pIgA transport to the apical plasma membrane as well as its extracellular secretion. These findings indicate that Rab11-FIP1 and Rab11-FIP5 serve as two indispensable components to facilitate pIgA transcytosis. The three members of class I Rab11-FIPs are not redundant genes because they have distinct roles in regulatory mechanisms during pIgA transcytosis. It has been estimated that as much as 3000 mg of sIgA is transported daily into the mucosal surfaces such as intestine lumen of an average adult, hence we believe that the trafficking of pIgR/pIgA complex should be strictly regulated by the class I Rab11-FIPs for constant secretion of pIgA. The difference in the function of the three class I Rab11-FIPs is also embodied in many other aspects, such as in tumors, the central nervous system, and diseases [50,51,52,53,54].

It appears that Rab proteins, as motor molecules, are responsible for regulating the trafficking process. Although Rab5a is defined as a marker of the early endosome, it does not always colocalize with EEA1 (early endosomal antigen 1) [12,55,56]. Rab11 is usually regarded as a recycling endosome marker, but it is also found in the early endosomes and Golgi apparatus [11]. According to previous studies, different Rab proteins, such as Rab4, Rab5, and Rab11, may locate in distinct domains of the same endosomes, comprising multiple and dynamic combinations in the same endosomes. Cargo moves through distinct domains of the endosomes, and different Rab domains recruit specific effector proteins to coordinate the transport process [13]. In this study, we found that Rab11a rapidly became a plasma membrane-bound form, colocalized with pIgA within ~10 min, and participated in almost the whole pIgA trafficking process. However, as the effectors of Rab11, Rab11-FIP1 and Rab11-FIP5 began to colocalize with Rab11a-positive endosomes containing pIgA at ~15 min after pIgA treatment (Figure 5C). We hypothesized multiple effectors of Rab11a are distributed in the different regions of cells and successively function in an auxiliary role, and that other effectors of Rab11a involved in pIgA trafficking within ~15 min might exist.

Ubiquitination modification plays a vital role in the trafficking of cargo proteins and signal transduction [33,34,35]. For example, K48-linked ubiquitination mainly mediates the degradation of substrates, and K63-linked ubiquitination promotes the activation of downstream signals or maintains the stability of target proteins [57,58]. In this study, pIgA transcytosis triggered polyubiquitination and upregulated expression of Rab11-FIP1 (Figure 6A and Figure S6A,B). Recently, it has been reported that the ubiquitin ligase RFFL mediates the ubiquitination of Rab11-FIP1 to regulate the integral function of the endocytic recycling compartment [59]. We found that RFFL knockdown had little effect on the extracellular secretion of pIgA in incompletely polarized Vero-pIgR cells, which might be attributed to the difference of cell species. However, TRIM21 knockdown impaired the transcytosis and extracellular secretion of pIgA (Figure 6E,F,H). Moreover, TRIM21 mediated the K11-linked polyubiquitination of Rab11-FIP1 and the K6-linked polyubiquitination of Rab11-FIP5 (Supplementary Figures S8A,B and S9A,B). Activated Rab11-FIP1 and Rab11-FIP5 promoted the trafficking of pIgR-pIgA complex to the Golgi apparatus. As TRIM21 also serves as a cytoplasmic Fc receptor, whether TRIM21 and pIgR additively recognize pIgA to promote its transcytosis in our incompletely polarized cell model needs further clarification. In addition, knockdown of TRIM21 did not significantly impair the trafficking of Rab11-FIP1 from the centrosome to Golgi apparatus or extracellular secretion of pIgA, implying that other E3 ubiquitin ligases or modification types of Rab11-FIP1 might exist and that multiple modifications cooperatively facilitate the activation of Rab11-FIP1 and transcytosis of pIgA. Our study reveals that TRIM21 can also mediate other types of ubiquitination beyond the K48- and K63-linked ubiquitin chains and may play more potent regulatory roles in the recognition and sorting of cargo proteins.

**Figure 7:**
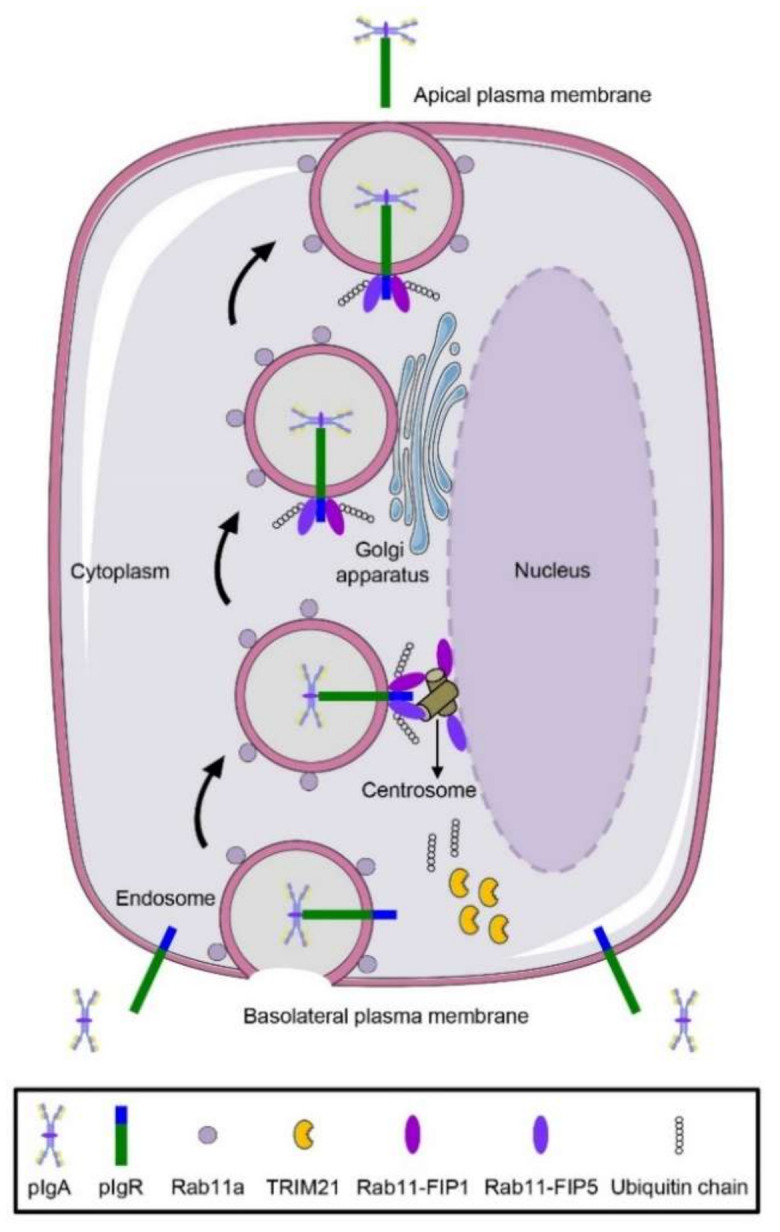
A working model on Rab11-FIP1- and Rab11-FIP5-mediated pIgA transcytosis in incompletely polarized cells. In our incompletely polarized cells model, pIgA is recognized and bound by pIgR upon pIgA treatment. Subsequently, the endocytic pIgR–pIgA complex is delivered by Rab11a-positive endosomes. The complex is first transported from the basolateral plasma membrane to the vicinity of the centrosome where Rab11-FIP1 and Rab11-FIP5 bind to it. During the trafficking process, TRIM21 mediates the K11-linked polyubiquitination of Rab11-FIP1 and the K6-linked polyubiquitination of Rab11-FIP5 to promote their activation. With the assistance of Rab11-FIP1 and Rab11-FIP5, pIgR–pIgA complex is further transferred to the apical plasma membrane via Golgi apparatus and then secreted.

In recent decades, studies related to pIgA transcytosis mainly focused on polarized MDCK cells. However, under pathological conditions, environmental enrichment can induce pIgA-dependent wound repair in a colon-tumor-bearing mice model [60]. These studies indicate that pIgA might be involved in wound healing and it is necessary for us to explore pIgA transcytosis process in incompletely polarized cells. Whether the transport process of pIgA exhibits differences between incomplete polarization and polarization conditions remains poorly investigated. In light of these analyses, we proposed that in an incompletely polarized cell model, pIgA was rapidly recognized and bound by pIgR upon pIgA treatment. Subsequently, the endocytic pIgR–pIgA complex was first transported from the basolateral plasma membrane to the vicinity of the centrosome, where Rab11-FIP1 and Rab11-FIP5 interacted with it. Meanwhile, TRIM21 mediated the K11-linked polyubiquitination of Rab11-FIP1 and the K6-linked polyubiquitination of Rab11-FIP5 to promote their activation. With the assistance of Rab11-FIP1 and Rab11-FIP5, the pIgR–pIgA complex was further transferred to the apical plasma membrane via Golgi apparatus and then secreted (Figure 7). Although the incompletely polarized cell model cannot represent real pathological conditions, our results provide a new perspective for elucidating the roles of pIgA transcytosis in the wound repair of the colon tumor model. The endocytosis of pIgR–pIgA might activate intracellular signal transduction and promote the nuclear translocation of some unknown transcription factors. These transcription factors induce the transcription and subsequent translation of Rab11-FIP1 and Rab11-FIP5, thereby facilitating Rab11-FIP1/Rab11-FIP5 activation and pIgA transcytosis.

## 4. Materials and Methods

### 4.1. Reagents, Antibodies and Cells

Protease inhibitor cocktail (TargetMol, Shanghai, China); biotin (Thermo Fisher, Waltham, MA, USA); Streptavidin Agarose (YeaSen Biotechnology, Shanghai, China); protein G Sepharose and Glutathione Sepharose (GE Healthcare, Uppsala, Sweden); polybrene (Millipore, Burlington, MA, USA); mouse monoclonal antibodies against Flag (RM1002), HA (RM1004), V5 (RM1006), and β-actin (RM2001) were purchased from Beijing Ray Antibody Biotech (Beijing, China); mouse monoclonal antibodies against pIgR (sc-374343), Rab11-FIP1 (sc-517228), ubiquitin (sc-8017) and Rab11a (sc-166523) were purchased from Santa Cruz Biotechnology (Dallas, TX, USA); anti-ubiquitin K11 linkage antibody (MABS107) was purchased from Sigma Aldrich (St. Louis, MO, USA); KDEL (ab12223) and HRP-conjugation Kit (ab102890) were purchased from Abcam (Cambridge, MA, USA); GM130 (610822) was purchased from BD Biosciences (Frankin Lakes, NJ, USA); Rab11-FIP1 (A9215), Rab11-FIP5 (A18142), γ-Tubulin (A9657) and TRIM21 (A1957) were purchased from ABclonal Technology (Wuhan, China); mouse IgA (0106-01), goat anti-mouse IgA (1040-01), goat anti-mouse IgA-AP (1040-04), goat anti-mouse IgA conjugated to Alexa Fluor 647 (1040-31) were purchased from SouthernBiotech (Birmingham, AL, USA); HRP-Streptavidin (405210) was purchased from BioLegend (San Diego, CA, USA). Goat anti-mouse IgG-HRP (115-035-164), goat anti-rabbit IgG-HRP (115-035-008) were purchased from Jackson (West Grove, PA, USA); Goat anti-rabbit IgG conjugated to Alexa Fluor 488 (R37116) and goat anti-mouse IgG conjugated to Alexa Fluor 594 (R37121) were purchased from Thermo Fisher (Waltham, MA, USA).

Caco-2 (ATCC HTB-37), Vero C1008 (ATCC CRL-1587), and human HEK293T (ATCC CRL-3216) cell lines were all obtained from ATCC (Manassas, VA, USA); Vero-pIgR cells were saved in our lab [61]. Mycoplasma was checked with no mycoplasma contamination present in any used cells.

### 4.2. Constructs

Mammalian expression plasmid pcDNA3.1 BirA*-HA (Addgene, 36047) was kindly provided by Professor Chaoyang Li (Guangzhou Medical University). The cDNA encoding human pIgR was amplified by PCR and cloned in between NheI and EcoRI of the vector pcDNA 3.1 BirA*-HA for mammalian expression. The cDNA encoding human pIgR and its mutants were amplified by PCR and cloned in between EcoRI and XbaI of the vector pRK-Flag for mammalian expression. The cDNA encoding human Rab11-FIP1, Rab11-FIP5 and its mutants were amplified by PCR and cloned in between FseI and AscI of the vector pCS2-HA for mammalian expression. The cDNA encoding human Rab11-FIP1 was amplified by PCR and cloned in between SalI and NotI of the vector pRK-GFP for mammalian expression. The cDNA encoding human Rab11-FIP5 was amplified by PCR and cloned in between SalI and NotI of the vector pRK-Cherry for mammalian expression. The cDNA encoding human TRIM21 was amplified by PCR and cloned in between SalI and NotI of the vector pRK-Flag for mammalian expression. The primers used for coding DNA sequence (CDS) amplification and tags location are listed in Supplementary Table S2. The pRK related vectors, ubiquitin plasmid and its mutants were kindly provided by Professor Yanyi Wang (Wuhan Institute of Virology, Chinese Academy of Sciences). Double-strand oligonucleotides corresponding to the target gene sequences were cloned into the pSuper retro RNAi plasmid. The following sequences were targeted for Rab11-FIP1 mRNA: #1 5′-CGCCTCTTTCCCAGTCCATGT-3′; #2 5′-GGAAGGACTTTCCTTTCTT-3′; #3 5′-GGAAGAAGGACGTGGCTAA-3′; the following sequences were targeted for pIgR mRNA: 5′-GAAGAGUUUGUUGCCACCATT-3′; the following sequences were targeted for Rab11-FIP5 mRNA: 5′-GAGCCGAGUGCUCAGGCUAAA-3′; the following sequences were targeted for TRIM21 mRNA: 5′-GATGCTCACAGGCTCCACGA-3′. These retro-shRNA plasmids were constructed by standard molecular biology techniques.

### 4.3. Affinity Purification of Biotinylated Proteins

HEK293T cells in 10 cm dishes were transfected with the indicated plasmids for 24 h in complete medium supplemented with 50 µM biotin. Cells from five confluent 10 cm cell culture dishes were collected and washed six times with ice cold PBS before lysis in 1 mL NP-40 lysis buffer (20 mM Tris-HCl, PH 7.5, 150 mM NaCl, 1 mM EDTA, 1% NP-40, protease inhibitor cocktail) on ice for 30 min. The lysates were centrifuged at 12,000 rpm for 15 min at 4 °C. Supernatant was added with 100 µL of Streptavidin Agarose beads. Bead-lysate mixtures were incubated with rocking at 4 °C overnight. Then the beads were washed twice for 8 min in 1 mL washing buffer 1 (2% SDS in ddH_2_O), once with washing buffer 2 (0.1% deoxycholate, 1% Triton X-100, 500 mM NaCl, 1 mM EDTA, and 50 mM Hepes, pH 7.5), once with washing buffer 3 (250 mM LiCl, 0.5% NP-40, 0.5% deoxycholate, 1 mM EDTA, and 10 mM Tris, pH 8.1) and twice with washing buffer 4 (50 mM Tris, pH 7.4, and 50 mM NaCl). For mass spectrometry analysis, beads should be washed twice in 50 mM NH_4_HCO_3_.

### 4.4. Coimmunoprecipitation and Immunoblotting Analyses

For transient transfection and coimmunoprecipitation experiments, HEK293T cells in 10 cm dishes were transfected with indicated plasmids for 24 h, the cells were lysed in l mL NP-40 lysis buffer on ice for 30 min. The lysates were centrifuged at 12,000 rpm for 15 min at 4 °C. The supernatant was respectively incubated with 0.5 µg of the indicated antibody or control IgG and 30 µL of Protein G Sepharose beads at 4 °C with overnight rotation. The Sepharose beads were washed three times with 1 mL lysis buffer containing 500 mM NaCl. The beads and cell lysates were boiled in the SDS-PAGE loading buffer for 10 min, separated by SDS-PAGE, and then transferred to PVDF membranes. The membranes were blocked for 1 h at room temperature in a Tris-buffered saline-Tween (0.05%) (TBST) solution with 5% non-fat dry milk and individually incubated with primary antibodies (1:1000 dilution) at 4 °C overnight. After subsequent washes with TBST, the membranes were then incubated with HRP-linked secondary antibodies (1:3000 dilution) at room temperature for 1 h and washed with TBST. The membranes were developed with enhanced chemiluminescence (ECL, Millipore, Burlington, MA, USA), as recommended by the manufacturer.

For endogenous coimmunoprecipitation experiments, Vero-pIgR (2 × 10^7^) cells in 24 mm diameter Transwell (Costar, Kennebunk, ME, USA, REF3450) cultured for three days were treated or not treated with 80 µg pIgA for 2 h. The cells were subjected to coimmunoprecipitation experiments as described above.

### 4.5. ELISA

The 96-well plate was coated with goat anti-mouse IgA unlabeled antibody (3 µg/mL) in carbonate-bicarbonate buffer at 4 °C overnight. After washing, they were blocked with 1% BSA at 37 °C for 2 h. Then, diluted samples were added to the plates for 2 h at 37 °C. After washing, goat anti-mouse IgA-AP was applied to the plates followed by substrate (p-nitrophenyl phosphate, Sigma Aldrich, St. Louis, MO, USA) coloring. ODs were read at 405 nm by an ELISA plate reader (Thermo Fisher, Waltham, MA, USA).

### 4.6. Confocal Microscopy

Cells cultured on 12 mm diameter Transwell (Costar, Kennebunk, ME, USA, REF3460) were fixed with 4% paraformaldehyde for 30 min at room temperature and permeabilized with 0.1% Triton X-100 in PBS for 20 min at room temperature. Then the cells were blocked with 1% BSA in PBS for 1 h at 37 °C. Cells were incubated with the indicated primary and secondary antibodies for 1 h at 37 °C. After washing with PBS, the nuclei were stained with DAPI. Cells were observed by Nikon confocal microscope with a 60× oil immersion objective lens. The image format was 1024 × 1024 pixels and processed with NIS-Elements Viewer software version 3.20. The *z*-axis series of optical sections were performed at 0.6 µm-thick sections. ImageJ v1.8.0 software, Coloc 2 plugin and Costes algorithm were used to determine the Pearson’s coefficient.

### 4.7. Transepithelial Resistance (TER) Measurements

TER was measured using Millicell-ERS-2 (Millipore, Burlington, MA, USA) for Transwell grown cells according to manufacturer’s instructions. The absolute TER values were determined according to manufacturer’s instructions.

### 4.8. Establishment of Stable Cell Lines

HEK293T cells were co-transfected with the indicated retroviral plasmids and two packaging plasmids (pGag-pol and pVSV-G) for 36 h. Then the medium was filtered with 0.45 µm filter and used to infect Vero-pIgR cells in the presence of polybrene (8 mg/mL). The stably silenced cells were selected with 4 µg/mL puromycin for 9 days. The knockdown efficiency of the stable cell lines was identified by immunoblotting. The RNAi sequence was listed as described above.

### 4.9. Ubiquitination Assays

Cells were lysed with lysis buffer (100 µL) containing 1% SDS and denatured at 95 °C for 5 min. The denatured lysates were diluted with NP-40 lysis buffer until the concentration of SDS was reduced to 0.1% followed by immunoprecipitation with the indicated antibodies.

### 4.10. In Vitro Ubiquitination Assay

The proteins were expressed with TNT Quick-coupled Transcription/Translation Systems kit (Promega, Madison, WI, USA) following instructions of the manufacturer. Ubiquitination was analyzed with a ubiquitination kit (Enzo Life Science, New York, NY, USA) following instructions of the manufacturer.

### 4.11. Quantitative Real-Time PCR

Total RNA was extracted from cells using RNAiso plus reagent (Vazyme, Nanjing, China) according to the manufacturer’s instructions. After reverse-transcription with oligo (dT) primer using a RevertAidTM First Strand cDNA Synthesis Kit (Vazyme, Nanjing, China), the samples were mixed with SYBR Green (Vazyme, Nanjing, China), and a primer mix to a final volume of 10 μL. Then the mix was subjected to real-time PCR analysis to measure mRNA expression levels of the tested genes. Data shown were the relative abundance of the indicated mRNAs normalized to GAPDH using the ΔΔCt method.

The qPCR primers were: 

Vero-Rab11FIP1-F: 5′-GCCAGAAAAAGTGCTGCTTCGTC-3′,

Vero-Rab11FIP1-R: 5′-GGGAAGGGTGAAGTTGACCTGG-3′;

Vero-Rab11FIP5-F: 5′-GAGTAGTTGGTTTGGCTTGAGAG-3′,

Vero-Rab11FIP5-R: 5′-TCAGGGCTATGCTTAGACTGGA-3′;

Vero-GAPDH-F: 5′-AAGGTCGGAGTCAACGGATT-3′,

Vero-GAPDH-R: 5′-CTCCTGGAAGATGGTGATGG-3′.

### 4.12. Statistical Analysis

For two samples, unpaired, two-tailed Student’s *t*-test was used for statistical analysis and the F test was performed to confirm that two populations have the same variances. For multiple comparisons, the non-parametric statistical Kruskal–Wallis one-way ANOVA was performed. Statistical differences were evaluated using GraphPad Prism Software version 8.0.

## Data Availability

The data presented in this study are available in the article and supplementary material.

## Supplementary Materials

The following are available online at https://www.mdpi.com/article/10.3390/ijms221910466/s1.
