# Supplementary material for: Rab11-FIP1 and Rab11-FIP5 Regulate pIgR/pIgA Transcytosis through TRIM21-Mediated Polyubiquitination
Source: Int J Mol Sci. 2021 Sep 28;22(19):10466. doi: 10.3390/ijms221910466 (PMC8508952; doi:10.3390/ijms221910466)
Supplement: Supplementary file 1 [file ijms-22-10466-s001.zip › ijms-1345119-supplementary.pdf]

Rab11-FIP1 and Rab11-FIP5 regulate pIgR/pIgA transcytosis through TRIM21-mediated polyubiquitination

Xuxu Fan[1,3], Dihan Zhou[1], Bali Zhao[2], Huijun Sha[1,3], Mengxue Li[1,3], Xian Li[1,3],
Jingyi Yang[2*], Huimin Yan[1,2,3*]

[1] Mucosal Immunity Research Group, State Key Laboratory of virology,
Wuhan Institute of Virology, Center for Biosafety Mega-Science,
Chinese Academy of Sciences,
Wuhan, 430071, China

[2]Vaccine and Immunology Research Center,
Translational Medical Research Institute,
Shanghai Public Health Clinical Center, Fudan University,
Shanghai, 201508, China

[3]University of Chinese Academy of Sciences,
Beijing, 100049, China

*To whom correspondence should be addressed:
Dr. Huimin Yan (hmyan@wh.iov.cn)
Dr. Jingyi Yang (jyyang875@163.com)

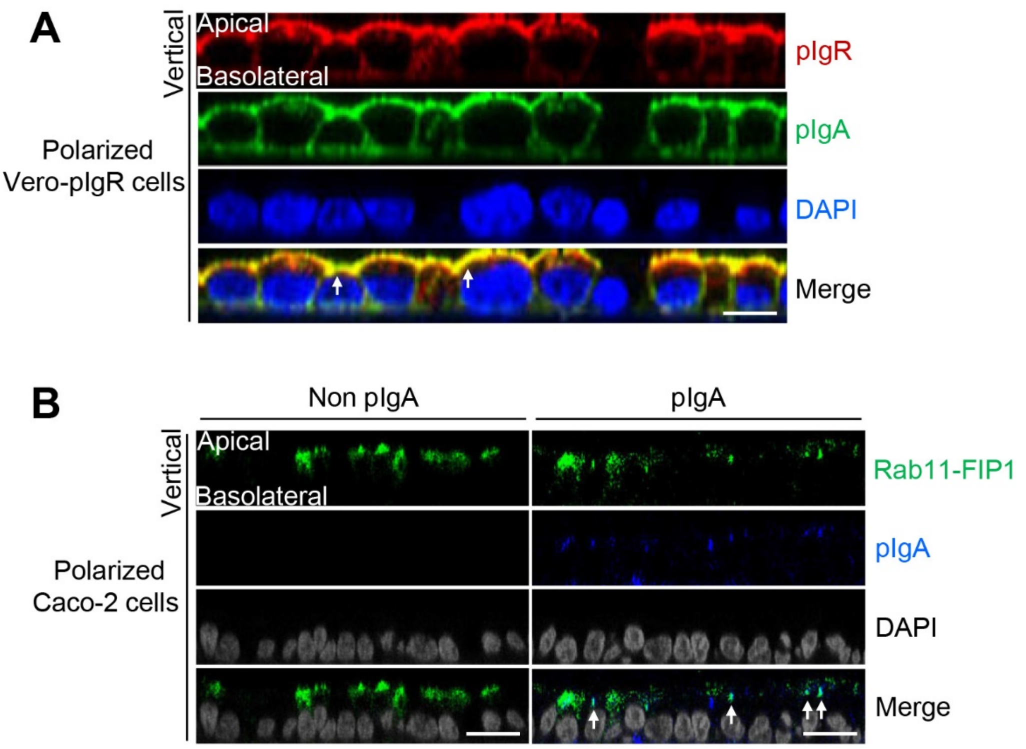

Figure S1. Colocalization of Rab11-FIP1 with pIgA was detected in polarized cells. (**A**) Colocalization of pIgR with pIgA. Vero-pIgR cells (1×10^5) were grown on 12 mm diameter Transwell (0.4 μm pore) for 6 days. An amount of 20 μg pIgA was added to the basal chamber for 1 h. The cells were fixed with 4% paraformaldehyde and stained with the indicated antibodies before observation by confocal microscopy. Scale bar: 20 μm. (**B**) Analysis of Rab11-FIP1 distribution in polarized Caco-2 cells. Caco-2 cells (1×10^5) were grown on 12 mm diameter Transwell (0.4 μm pore) for 7 days. An amount of 20 μg pIgA was added or not added to the basal chamber for 1 h. The cells were fixed with 4% paraformaldehyde and stained with the indicated antibodies before observation by confocal microscopy. Scale bar: 20 μm. Data of (**A**-**B**) are representative of three independent experiments.

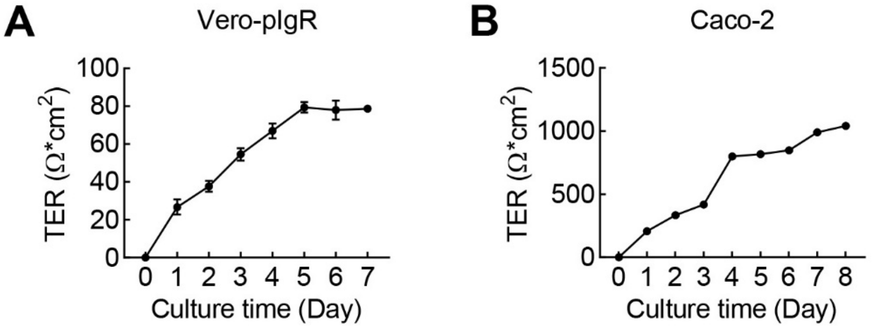

Figure S2. The measurement of TER in Transwell system. (**A**, **B**) Transepithelial electrical resistance (TER) of cultured monolayers. TER (Ω *cm^2) was measured in filter grown monolayers of Vero-pIgR or Caco-2 cells at the indicated times. Data of (**A**-**B**) are representative of three independent experiments.

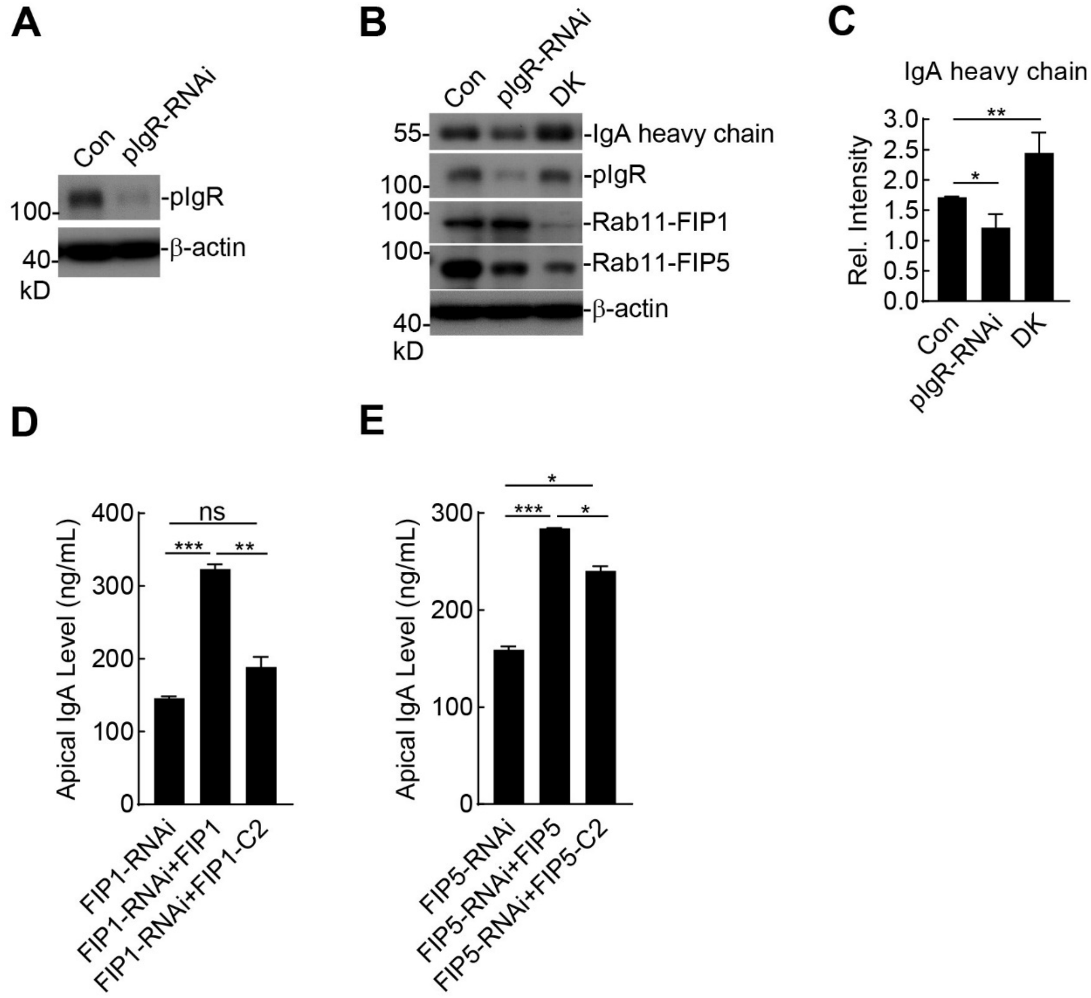

Figure S3. Double knockdown of Rab11-FIP1 and Rab11-FIP5 induced intracellular accumulation of pIgA. (**A**) Knockdown efficiency of pIgR. Vero-pIgR cells were transduced with the control or pIgR-RNAi plasmids. The knockdown efficiency of pIgR was detected by immunoblotting analysis. (**B**) Analysis of the intracellular levels of pIgA in Rab11-FIP1 and Rab11-FIP5 double-knockdown cells. The indicated Vero-pIgR cells (1×10^5) were grown on Transwell for 3 days. An amount of 20 μg pIgA was added to the basal chamber for 4 h. Cells were collected for immunoblotting analysis with the indicated antibodies. (**C**) Relative intensity of pIgA bands (normalized to the intensity of β-actin bands). **(D**, **E)** Effects of reconstitution of Rab11-FIP1 knockdown and Rab11-FIP5 knockdown cells with Rab11-FIP1 or Rab11-FIP5 mutant plasmids on extracellular secretion of pIgA. The indicated knockdown Vero-pIgR cells (1×10^5) were transduced with the indicated plasmids and were grown on Transwell for 3 days. An amount of 20 μg pIgA was added to the

basal chamber for 4 h. The supernatant was collected for IgA analysis by ELISA. Data were analyzed by unpaired, two-tailed Student's *t*-test. Graphs show mean ± SD; n = 3. ns, not significant, *$P < 0.05$, **$P < 0.01$, ***$P < 0.001$. Data of (**A**-**E**) are representative of three independent experiments.

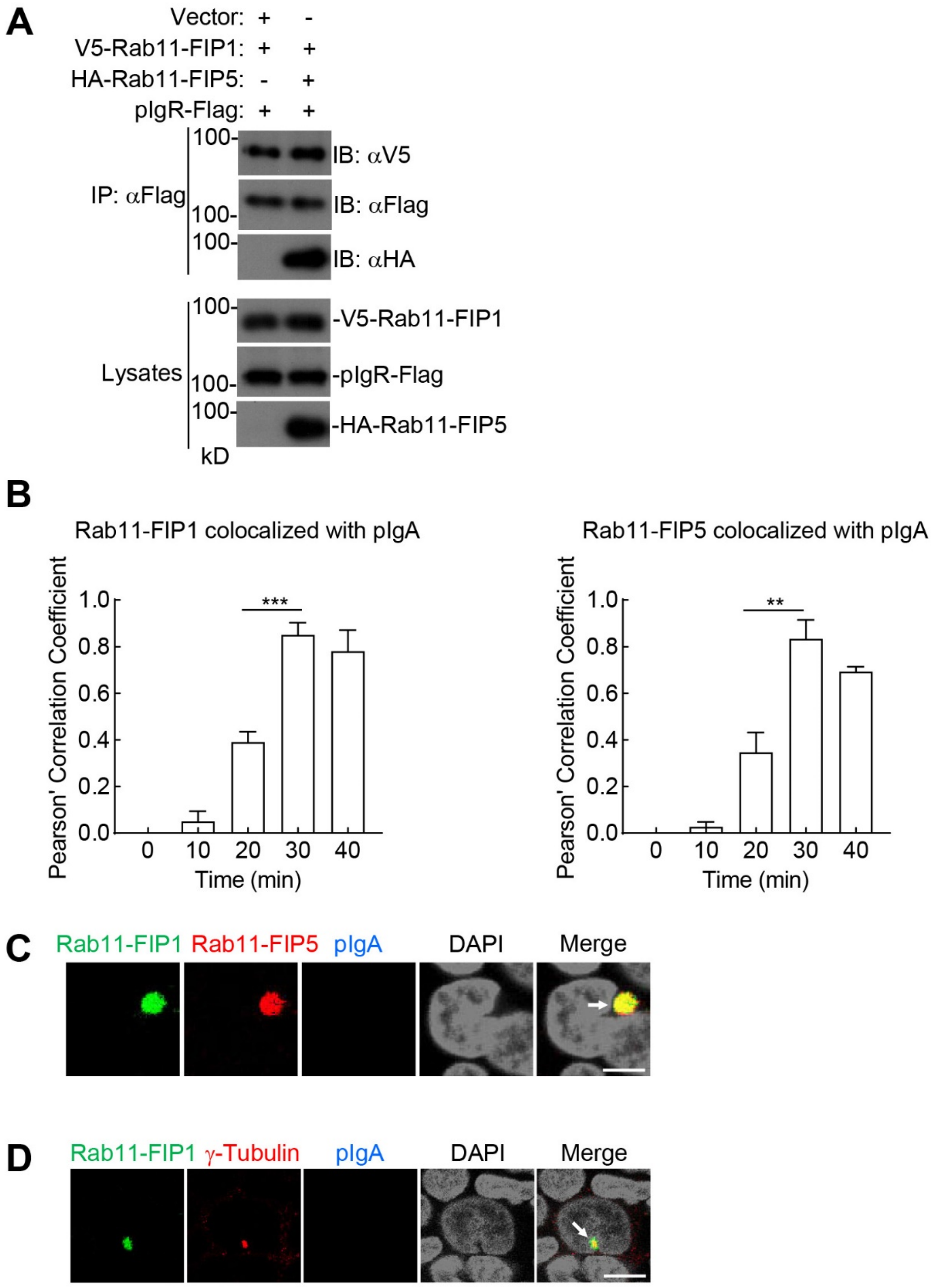

Figure S4. Colocalization of Rab11-FIP1 with Rab11-FIP5 was detected in incompletely polarized Caco-2 cells. (**A**) HEK293T cells (2×10^6) were co-transfected with the indicated plasmids for 24 h. Coimmunoprecipitation and immunoblot analyses were performed with the indicated antibodies. (**B**) Quantitative analysis of colocalization of Rab11-FIP1 and Rab11-FIP5 with pIgA in Vero-pIgR cells of Figure 4E. Statistical analysis was based on colocalization images (covering

dozens of cells) using the ImageJ software. (**C**)Colocalization of Rab11-FIP1 and Rab11-FIP5 in incompletely polarized Caco-2 cells without pIgA transcytosis. Caco-2 cells (1×10^5) were grown on Transwell for 3 days. Cells were fixed with 4% paraformaldehyde and stained with the indicated antibodies before observation by confocal microscopy. Scale bar: 10 μm. (**D**) Analysis of Rab11-FIP1 distribution in incompletely polarized Caco-2 cells without pIgA transcytosis. Caco-2 cells (1×10^5) were grown on Transwell for 3 days. Cells were fixed with 4% paraformaldehyde and stained with the indicated antibodies before observation by confocal microscopy. Scale bar: 10 μm. Data of (**A**, **C**, **D**) are representative of three independent experiments. Data of (**B**) were analyzed by unpaired, two-tailed Student's *t*-test. Graphs show mean ± SD; $n = 3$. $^{**}P < 0.01$, $^{***}P < 0.001$.

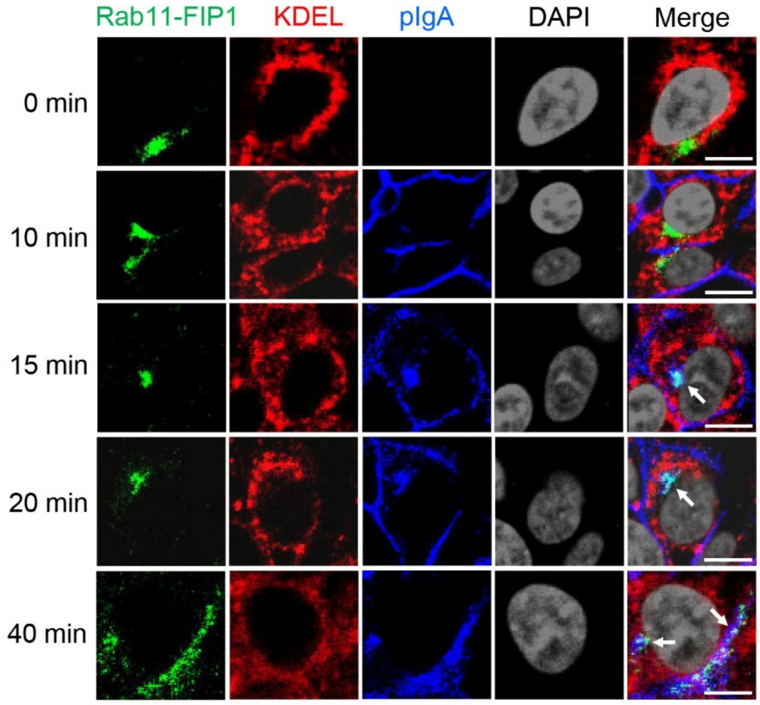

Figure S5. Colocalization of Rab11-FIP1, pIgA with KDEL was detected. Vero-pIgR cells (1×10^5) were grown on Transwell for 3 days. An amount of 20 μg pIgA was added or not added to the basal chamber for 10 min at 37 °C and cells were then washed for three times. Subsequently, cells were cultivated at 37 °C and harvested at the indicated time points. Finally, cells were fixed with 4% paraformaldehyde and stained with the indicated antibodies before observation by confocal microscopy. Scale bar: 10 μm. Data are representative of three independent experiments.

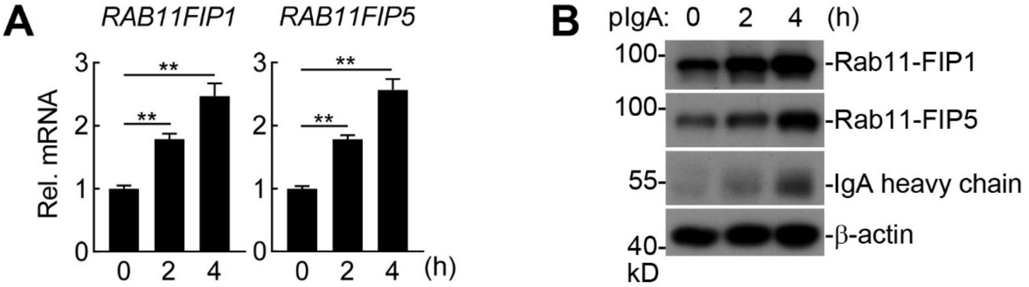

Figure S6. The mRNA and protein levels of Rab11-FIP1 and Rab11-FIP5 were detected during pIgA transcytosis. (**A, B**) Effects of pIgA transcytosis on the transcription and translation of Rab11-FIP1 and Rab11-FIP5. Vero-pIgR cells (1×10^5) were grown on Transwell for 3 days. An amount of 20 μg pIgA was added or not added to the basal chamber for the indicated time points. Cells were harvested for qPCR and immunoblotting analyses with the indicated antibodies. Data of (**A**) were analyzed by unpaired, two-tailed Student's t-test. Graphs show mean ± SD; n = 3. **P < 0.01. Data of (**A-B**) are representative of three independent experiments.

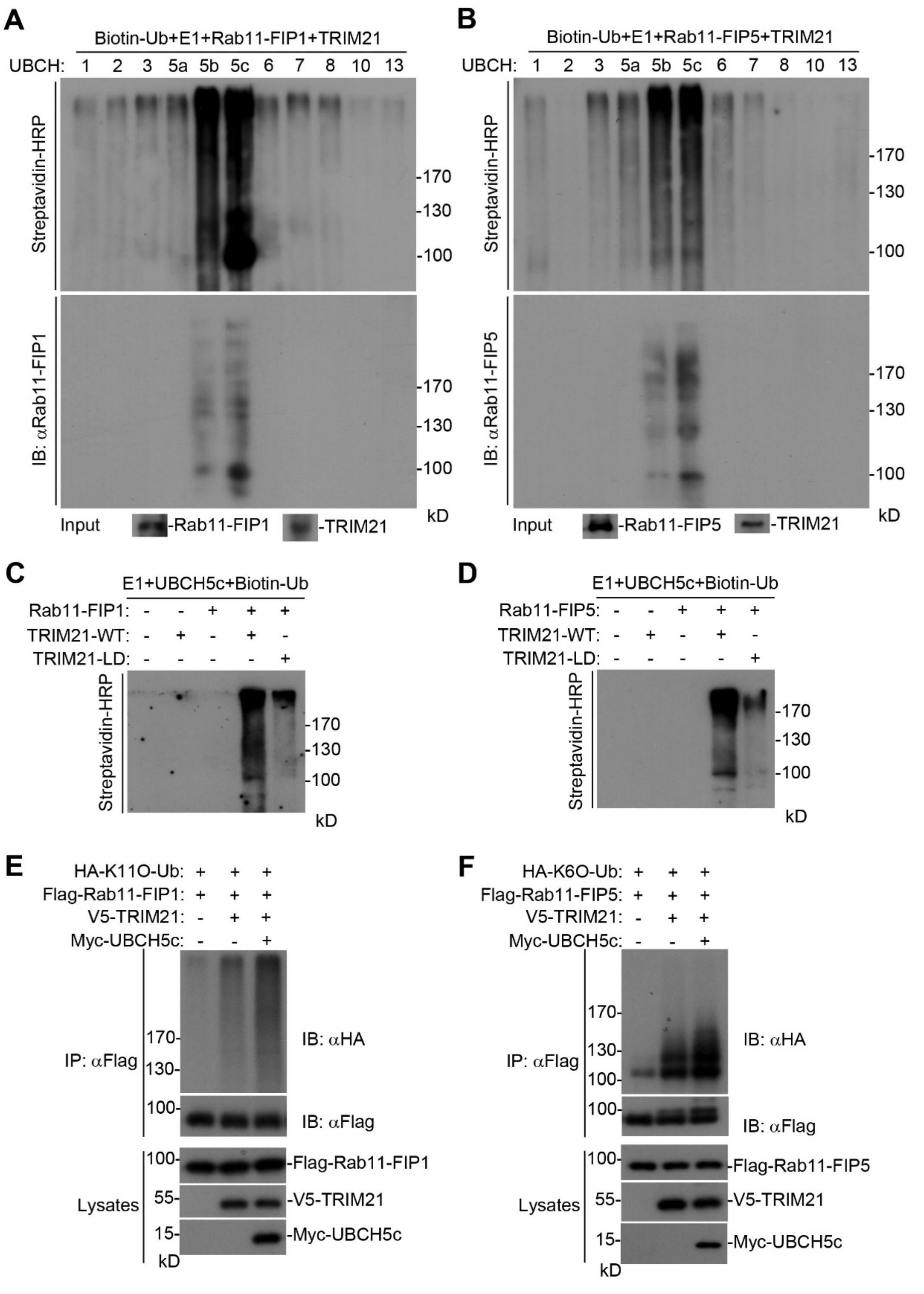

Figure S7. TRIM21 mediated polyubiquitination of Rab11-FIP1 and Rab11-FIP5 was detected *in vitro*. (**A**-**D**) Rab11-FIP1, Rab11-FIP5, TRIM21, and TRIM21-LD were translated *in vitro*, and then E1, the indicated E2s, and biotin-ubiquitin were added for *in vitro* ubiquitination assays. The ubiquitin-conjugated proteins were detected by immunoblotting with streptavidin-HRP and anti-Rab11-FIP1 and anti-Rab11-FIP5

antibodies (**A**-**B**). The expression levels of the related proteins were examined by immunoblotting with the indicated antibodies (**C**-**D**). (**E**-**F**) HEK293T cells (2×10^6) were co-transfected with the indicated plasmids for 24 h. Ubiquitination assays were performed with the indicated antibodies. K11O: all lysine residues within ubiquitin except K11 were mutated to arginine. K6O: all lysine residues within ubiquitin except K6 were mutated to arginine. Data of (**A**-**F**) are representative of three independent experiments.

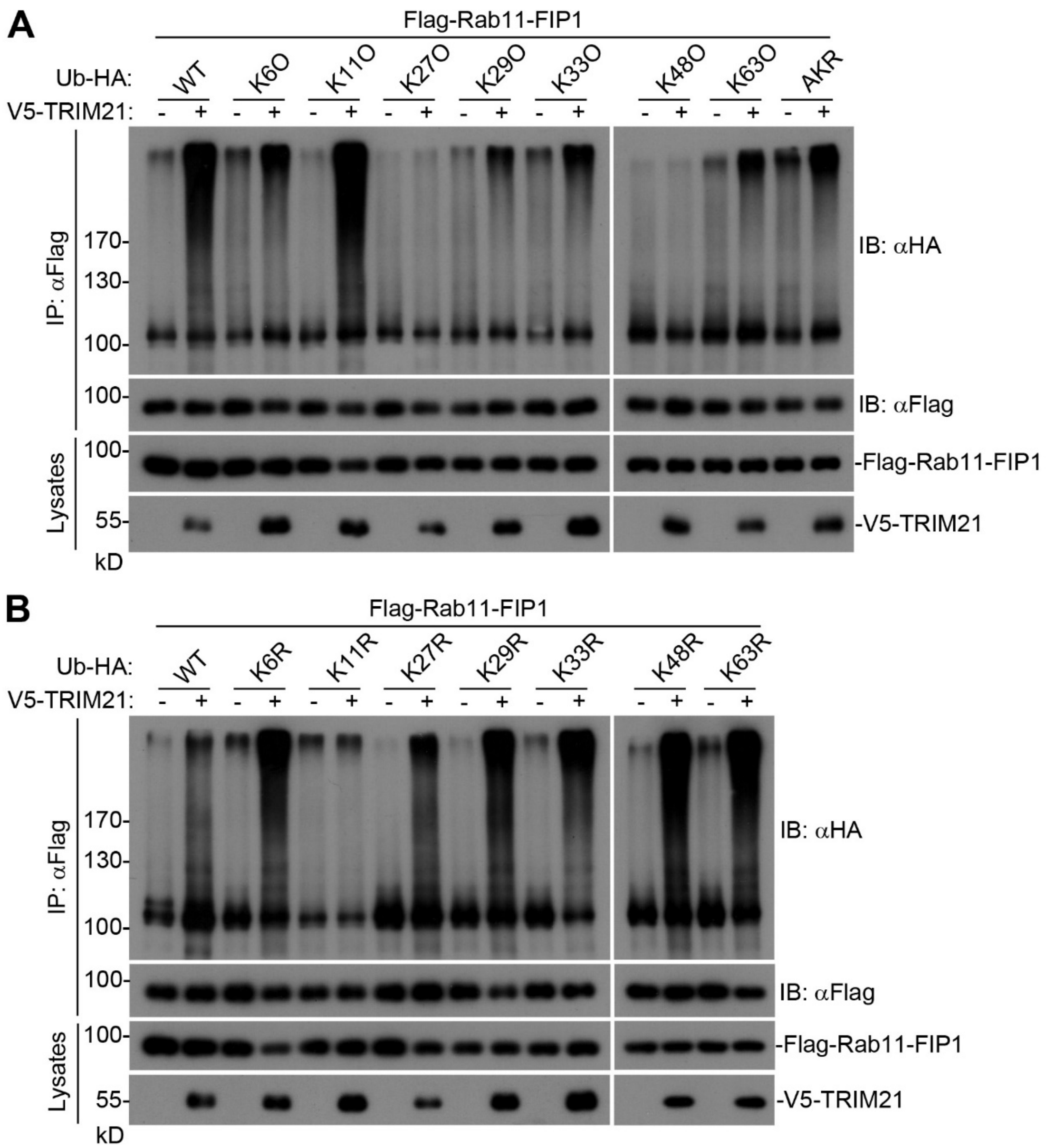

Figure S8. TRIM21 mediated K11-linked polyubiquitination of Rab11-FIP1 was detected. (**A**, **B**) HEK293T cells (2×10^6) were co-transfected with the indicated plasmids for 24 h. Ubiquitination assays were performed with the indicated antibodies. All experiments were performed three times with the similar results. K11O: all lysine residues within ubiquitin except K11 were mutated to arginine. K11R: only lysine residue K11 within ubiquitin was mutated to arginine.

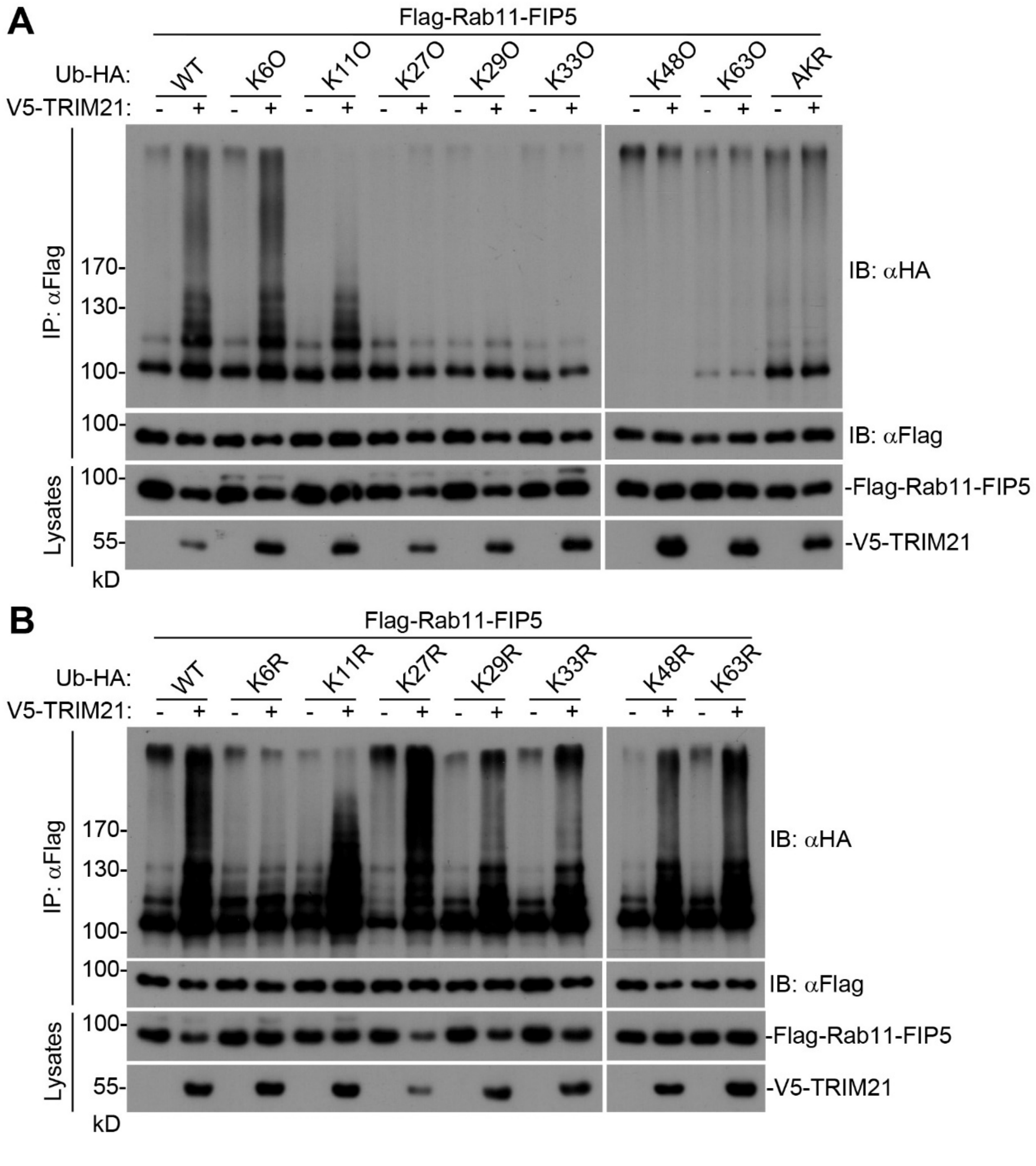

Figure S9. TRIM21 mediated K6-linked polyubiquitination of Rab11-FIP5 was detected. (**A**, **B**) HEK293T cells (2×10^6) were co-transfected with the indicated plasmids for 24 h. Ubiquitination assays were performed with the indicated antibodies. K6O: all lysine residues within ubiquitin except K6 were mutated to arginine. K6R: only lysine residue K6 within ubiquitin was mutated to arginine. Data of (**A**-**B**) are representative of three independent experiments.

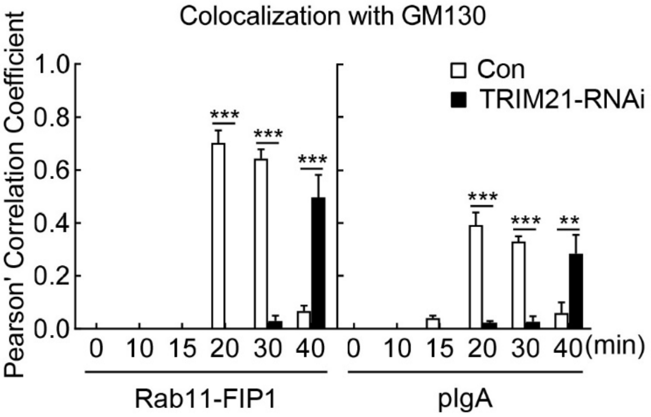

Figure S10. Quantitative analysis of colocalization of Rab11-FIP1 and pIgA with GM130 in wild-type and TRIM21-knockdown cells. Quantitative analysis of colocalization of Rab11-FIP1 and pIgA with GM130 in wild-type and TRIM21-knockdown cells. Statistical analysis was based on colocalization images (covering dozens of cells) using the ImageJ software. Data were analyzed by unpaired, two-tailed Student's *t*-test. Graphs show mean ± SD; $n = 3$. $^{**}P < 0.01$, $^{***}P < 0.001$.

Supplementary Table S1.

Accession	Gene	# PSMs_PIGR	# PSMs_PIGR-biotin
Q04721	NOTCH2	9	69
Q13085	ACACA	38	60
P11498	PC	26	35
P01833	PIGR	1	35
P05165	PCCA	9	18
F8VVR8	MYRFL	#N/A	9
Q96RQ3	MCCC1	4	8
O95125	ZNF202	2	7
P04264	KRT1	2	5
P35908	KRT2	#N/A	5
P07437	TUBB	1	3
P24043	LAMA2	1	3
A0A2U3TZI	EEF1A2	#N/A	3
Q14980	NUMA1	#N/A	3
Q06830	PRDX1	1	2
E7EQ64	PRSS1	1	2
P21333	FLNA	#N/A	2
Q6WKZ4	RAB11FIP1	#N/A	2
P13645	KRT10	#N/A	2
A5A3E0	POTEF	1	1
A0A0U1RR	SEC24C	4	1
A0A0G2JPF	SCRIB	#N/A	1
Q9Y2J2	EPB41L3	#N/A	1
P62913	RPL11	#N/A	1
J3KPF3	SLC3A2	#N/A	1
F5GXV7	NBEA	#N/A	1
P49411	TUFM	#N/A	1
P68363	TUBA1B	#N/A	1
P21912	SDHB	#N/A	1
G8JLB6	HNRNPH1	#N/A	1
P35527	KRT9	1	#N/A
P62805	HIST1H4A	1	#N/A
A2VDJ0	TMEM131L	1	#N/A
Q8NEZ4	KMT2C	2	#N/A
Q6P1N9	TATDN1	1	#N/A
P51884	LUM	1	#N/A
Q3SY77	UGT3A2	1	#N/A

Table S1. The list of proteins identified by mass spectrometry. Immunoprecipitates generated in Figure 1A were analyzed by TripleTOF 5600 mass spectrometry. The numbers in this table represented the abundance of protein in corresponding immunoprecipitates. #N/A: not detected.

Supplementary Table S2.

Vector	Tag site	Primer name	Primer sequence (5'-3')
pcDNA3.1 BirA*-HA	C	pIgR-NheI-F	CTAGCTAGCATGCTGCTCTTCGTGCTCACCT
		pIgR-EcoRI-R	TATTGAATTCGGCTTCCTGGGGGCCGTCCT
pRK-Flag	C	pIgR-F	GTTCTATCGATTGAATTCATGCTGCTCTTCGTGCT CACC
		pIgR-R	GTAGTCACTAGTTCTAGAGGCTTCCTGGGGGCCG TCCTG
pRK-Flag	C	pIgR 1-661aa-F	GTTCTATCGATTGAATTCATGCTGCTCTTCGTGCT CACC
		pIgR 1-661aa-R	GTAGTCACTAGTTCTAGAGGCCACCCCCACAGCC ACGGC
pRK-Flag	C	pIgR 639-764aa-F	GTTCTATCGATTGAATTCATGGCGCTGGTCTCCAC CCTG
		pIgR 639-764aa-R	GTAGTCACTAGTTCTAGAGGCTTCCTGGGGGCCG TCCTG
pCS2-HA	N	Rab11-FIP1-F	CTTGAATTCAGGTCAGGGCCGGATGTCCCTAATG GTCTCG
		Rab11-FIP1-R	ACTCACTATAGTTCTAGAGGCGCGTTAGGAAAGT CCTTCCTT
pCS2-HA	N	Rab11-FIP1 1-108aa-F	TACCAGACTACGCTGGCCGGCCGATGTCCCTAAT GGTCTCGG
		Rab11-FIP1 1-108aa-R	TCACTATAGTTCTAGAGGCGCGCCTTACTCGGCG CGGCCCAG
pCS2-HA	N	Rab11-FIP1 109-576aa-F	TACCAGACTACGCTGGCCGGCCGGTGGACCTGCG GGATCTGC
		Rab11-FIP1 109-576aa-R	GACTCACTATAGTTCTAGAGGCGCGCCTTACATCA TGACCTCATTGTTC
pCS2-HA	N	Rab11-FIP1 109-649aa-F	ATGTACCAGACTACGCTGGCCGGCCGGTGGACCT GCGGGATCTGCACC
		Rab11-FIP1 109-649aa-R	GACTCACTATAGTTCTAGAGGCGCGCCTTACATCT TTCCTGCTTTTTT
pCS2-HA	N	Rab11-FIP5-F	TGTACCAGACTACGCTGGCCGGCCGATGGCCCTG GTGCGGGGCGCGGAG
		Rab11-FIP5-R	ACTCACTATAGTTCTAGAGGCGCGCCCTATTTGGG GGGGCCCGGGGGGAT
pRK-GFP	N	GFP-Rab11-FIP1-F	CGAGCTGTACAAGGCGTCGACCATGTCCCTAATG GTCTCGGC
		GFP-Rab11-FIP1-R	CTGCGCCTGCAGGTCGCGGCCGCTTACATCTTTC CTGCTTTTTT
pRK-Cherry	N	Cherry-Rab11-FIP5-F	TTAGAATTCATGGCCCTGGTGCGGGGCGCGGA

		Cherry-Rab11-FIP5-R	TTATCTAGATTTGGGGGGGCCCGGGGGGAT
pRK-Flag	N	TRIM21-F	GATGACAAGGGGTCGACCATGGCTTCAGCAGCACGCTT
		TRIM21-R	CTGCAGGTCGCGGCCGCTCAATAGTCAGTGGATCCTT
